# Author Correction: Chiral recognition and enantiomer excess determination based on emission wavelength change of AIEgen rotor

**DOI:** 10.1038/s41467-020-16302-9

**Published:** 2020-05-05

**Authors:** Ming Hu, Ying-Xue Yuan, Weizhou Wang, Dong-Mi Li, Hong-Chao Zhang, Bai-Xing Wu, Minghua Liu, Yan-Song Zheng

**Affiliations:** 1 Key Laboratory of Material Chemistry for Energy Conversion and Storage, Ministry of Education, School of Chemistry and Chemical Engineering, Huazhong University of Science and Technology, Wuhan, 430074 China; 2 College of Chemistry and Chemical Engineering, and Henan Key Laboratory of Function-Oriented Porous Materials, Luoyang Normal University, Luoyang, 471934 China; 3 Beijing National Laboratory for Molecular Science (BNLMS), CAS Key Laboratory of Colloid Interface and Chemical Thermodynamics, Institute of Chemistry, Chinese Academy of Sciences, Beijing, 100190 China

**Keywords:** Fluorescent probes, Sensors, Self-assembly, Sensors and biosensors

Correction to: *Nature Communications* 10.1038/s41467-019-13955-z, published online 9 January 2020

The original version of this Article contained an error in the author affiliations.

Affiliations 2 and 3 were inadvertently switched.

This has now been corrected in both the PDF and HTML versions of the Article.

