# Supplementary material for: Chiral recognition and enantiomer excess determination based on emission wavelength change of AIEgen rotor
Source: Nat Commun. 2020 Jan 9;11:161. doi: 10.1038/s41467-019-13955-z (PMC6952378; doi:10.1038/s41467-019-13955-z)
Supplement: Supplementary file 1 — Supplementary Information [file 41467_2019_13955_MOESM1_ESM.pdf]

Supporting Information

Chiral Recognition and Enantiomer Excess Determination Based on Emission Wavelength Change of AIEgen Rotor

Ming Hu,[1] Ying-Xue Yuan,[1] Hong-Chao Zhang,[1] Bai-Xing Wu,[1] Yan-Song Zheng*,[1]
Minghua Liu,[2]
Weizhou Wang,[3] Dong-Mi Li,[3]

[1] Key Laboratory of Material Chemistry for Energy Conversion and Storage, Ministry of Education, School of Chemistry and Chemical Engineering, Huazhong University of Science and Technology, Wuhan 430074, China. E-mail: zyansong@hotmail.com
[2] Beijing National Laboratory for Molecular Science (BNLMS), CAS Key Laboratory of Colloid Interface and Chemical Thermodynamics, Institute of Chemistry, Chinese Academy of Sciences, Beijing 100190, China
[3] College of Chemistry and Chemical Engineering, Luoyang Normal University, Luoyang 471022, China

Supplementary Figures

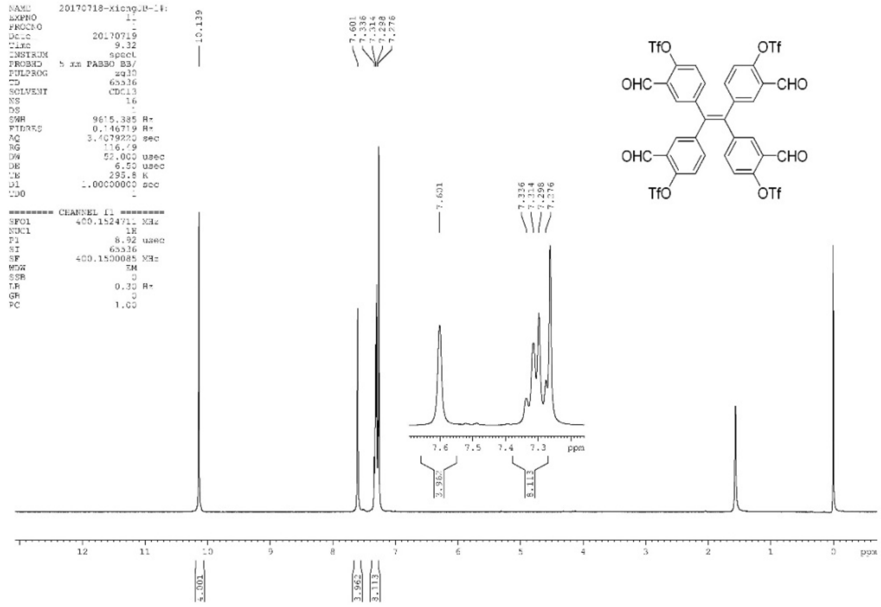

Supplementary Figure 1. ^{1}H NMR spectrum of compound **2** in $CDCl_3$.

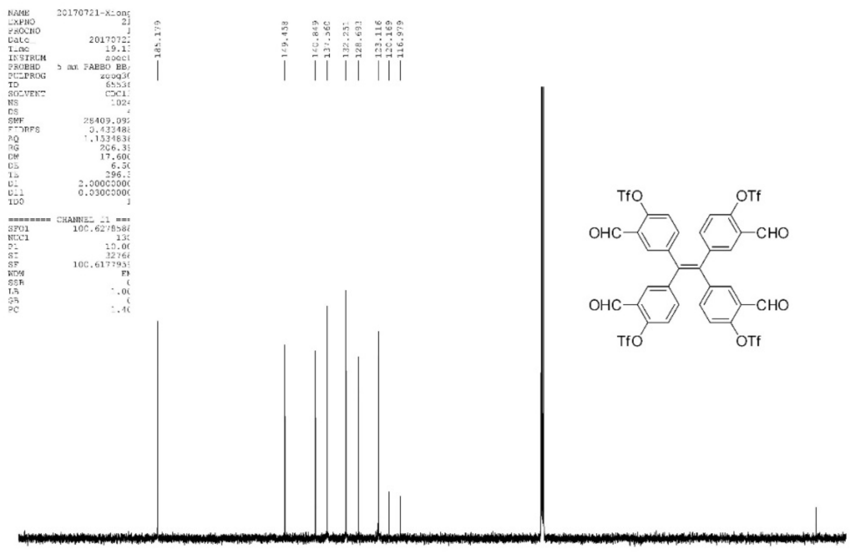

Supplementary Figure 2. ^{13}C NMR spectrum of compound **2** in $CDCl_3$.

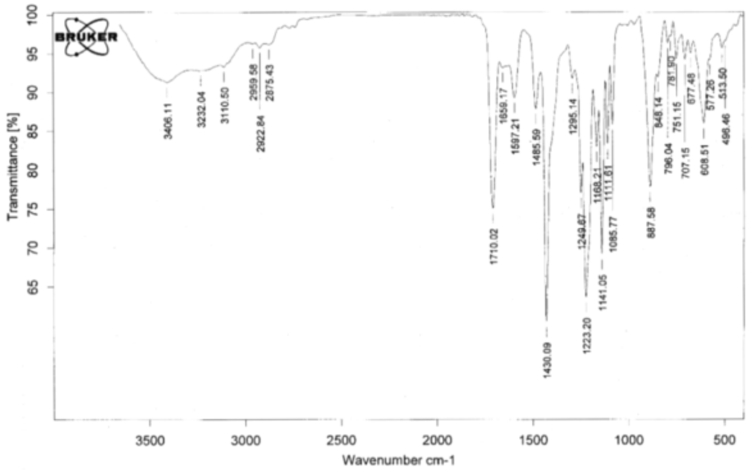

Supplementary Figure 3. IR spectrum of compound **2**.

Mass Spectrum List Report

Analysis Info

Analysis Name	D:\Data\ZhengYS\zheng-xiong-20170721-1.d	Acquisition Date	7/21/2017 9:50:50 AM
Method	tune_low.m	Operator	BDAL@DE
Sample Name	zheng-xiong-20170721-1	Instrument / Ser#	micrOTOF 10401
Comment			

Acquisition Parameter

Source Type	ESI	Ion Polarity	Positive	Set Nebulizer	0.3 Bar
Focus	Active			Set Dry Heater	200 °C
Scan Begin	50 m/z	Set Capillary	4500 V	Set Dry Gas	4.0 l/min
Scan End	2200 m/z	Set End Plate Offset	-500 V	Set Divert Valve	Waste

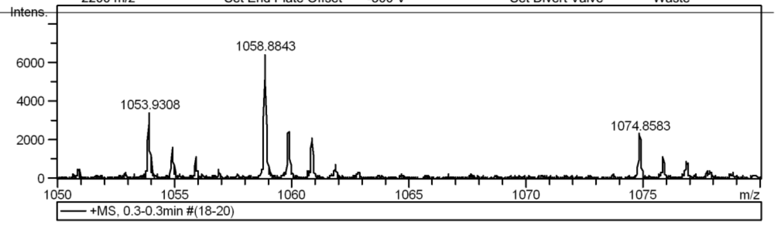

#	m/z	Res.	S/N	I	FWHM
1	1053.9308	12504	26.6	3331	0.0843
2	1054.9342	14109	12.4	1543	0.0748
3	1055.9334	11372	8.5	1055	0.0929
4	1058.8843	11082	51.5	6450	0.0956
5	1059.8870	9399	19.6	2418	0.1128
6	1060.8801	10996	16.7	2059	0.0965
7	1061.8728	10564	5.2	644	0.1005
8	1074.8583	10356	19.5	2324	0.1038
9	1075.8634	12849	9.0	1075	0.0837
10	1076.8464	9449	7.1	842	0.1140

Supplementary Figure 4. HRMS spectrum of compound **2**.

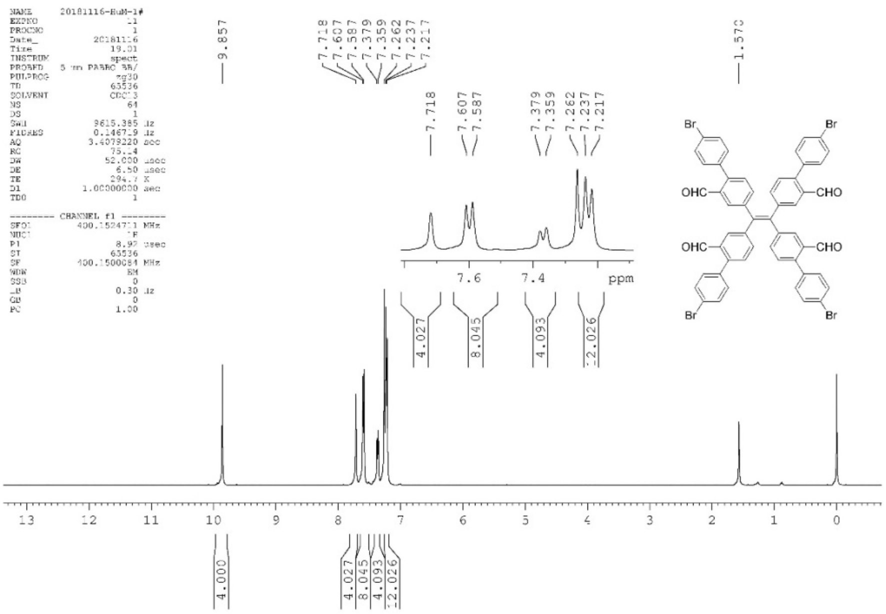

Supplementary Figure 5. ^{1}H NMR spectrum of compound **3** in $CDCl_3$.

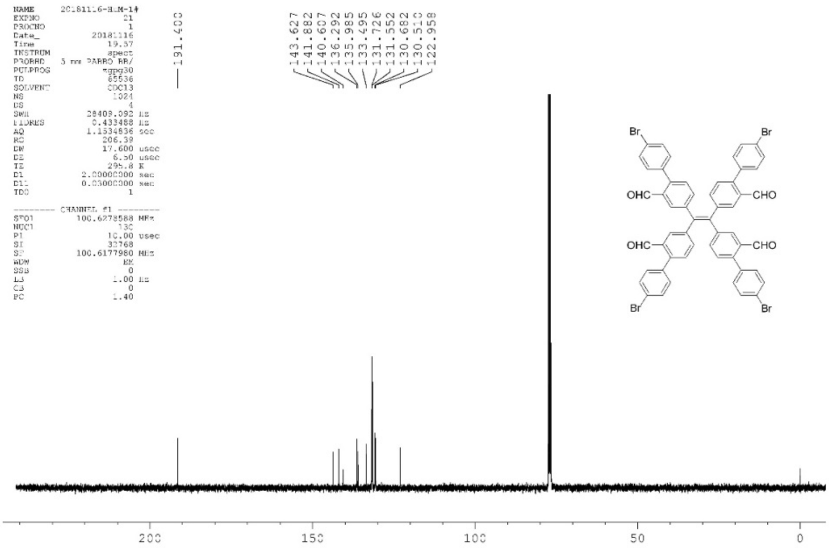

Supplementary Figure 6. ^{13}C NMR spectrum of compound **3** in $CDCl_3$.

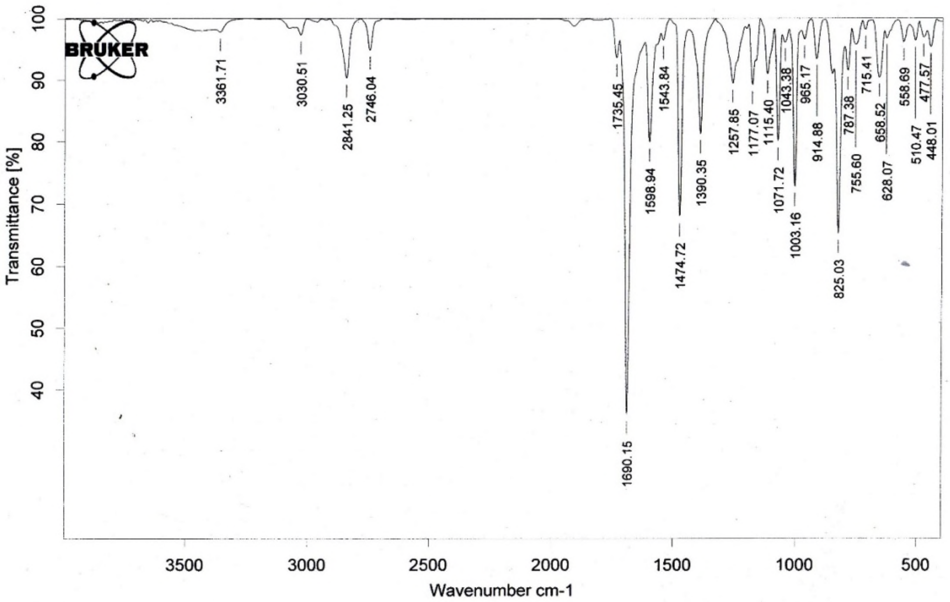

Supplementary Figure 7. IR spectrum of compound **3**.

Mass Spectrum List Report

Analysis Info

Analysis Name	D:\Data\ZhengYS\ZHENG-hum20190425-1.d	Acquisition Date	4/25/2019 10:10:46 AM
Method	tune_wide.m	Operator	BDAL@DE
Sample Name	ZHENG-hum20190425-1	Instrument / Ser#	micrOTOF 10401
Comment			

Acquisition Parameter

Source Type	ESI	Ion Polarity	Positive	Set Nebulizer	0.3 Bar
Focus	Active			Set Dry Heater	180 °C
Scan Begin	50 m/z	Set Capillary	4500 V	Set Dry Gas	4.0 l/min
Scan End	3000 m/z	Set End Plate Offset	-500 V	Set Divert Valve	Waste

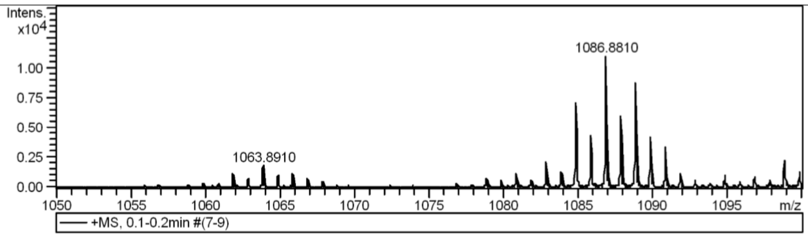

#	m/z	Res.	S/N	I	FWHM
1	1061.8953	13322	11.3	1167	0.0797
2	1062.9008	12012	7.0	724	0.0885
3	1063.8910	15061	17.1	1833	0.0706
4	1065.8939	11116	11.0	1142	0.0959
5	1084.8822	11605	65.0	7003	0.0935
6	1084.8822	11605	65.0	7003	0.0935
7	1086.8810	12266	101.0	10923	0.0886
8	1087.8869	12055	54.4	5893	0.0902
9	1088.8798	13094	80.2	8700	0.0832
10	1089.8840	12890	38.2	4151	0.0846
11	1090.8828	14661	30.4	3313	0.0744

Supplementary Figure 8. HRMS spectrum of compound **3**.

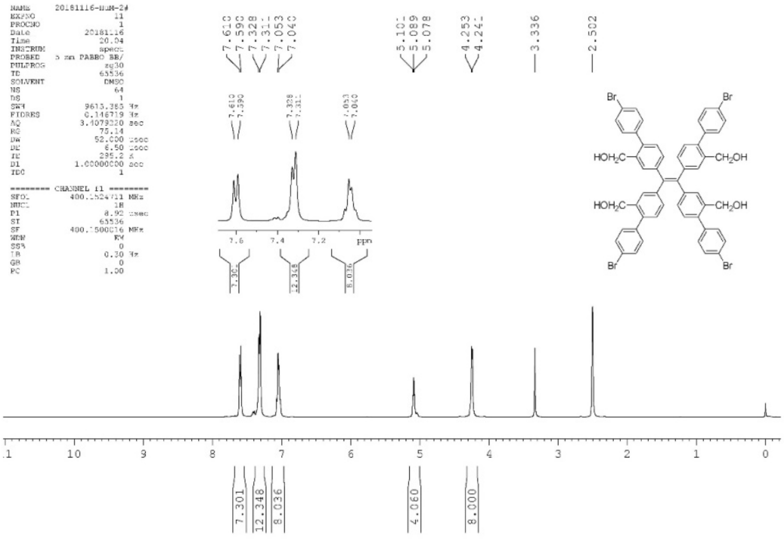

Supplementary Figure 9. ^{1}H NMR spectrum of compound **4** in DMSO-d$_6$.

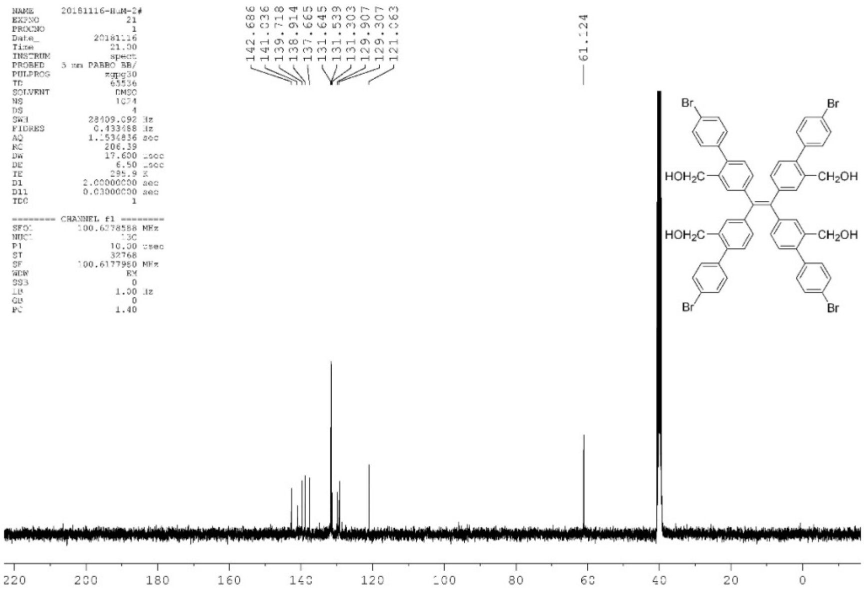

Supplementary Figure 10. ^{13}C NMR spectrum of compound **4** in DMSO-d$_6$.

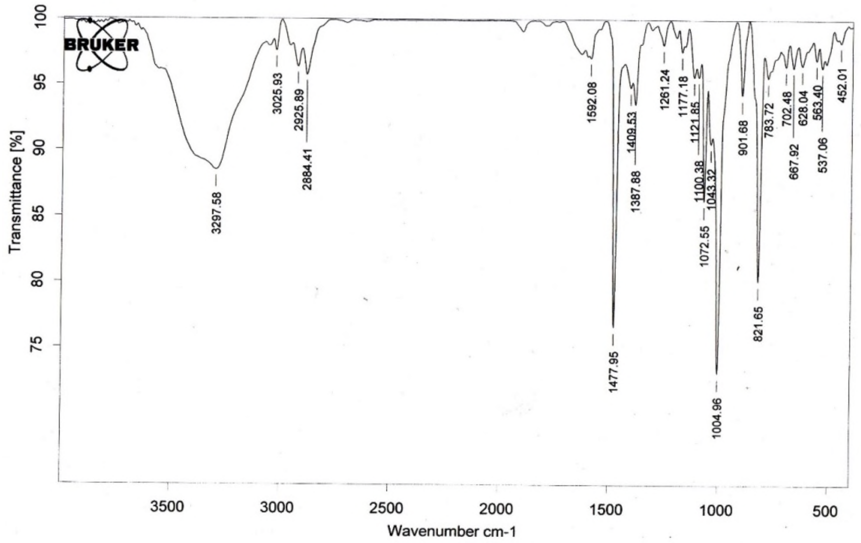

Supplementary Figure 11. IR spectrum of compound **4**.

Mass Spectrum List Report

Analysis Info

Analysis Name	D:\Data\ZhengYS\zheng-huming20190428.d	Acquisition Date	4/28/2019 3:08:20 PM
Method	tune_wide.m	Operator	BDAL@DE
Sample Name	zheng-huming20190428	Instrument / Ser#	micrOTOF 10401
Comment			

Acquisition Parameter

Source Type	ESI	Ion Polarity	Positive	Set Nebulizer	0.3 Bar
Focus	Active			Set Dry Heater	180 °C
Scan Begin	50 m/z	Set Capillary	4500 V	Set Dry Gas	4.0 l/min
Scan End	3000 m/z	Set End Plate Offset	-500 V	Set Divert Valve	Waste

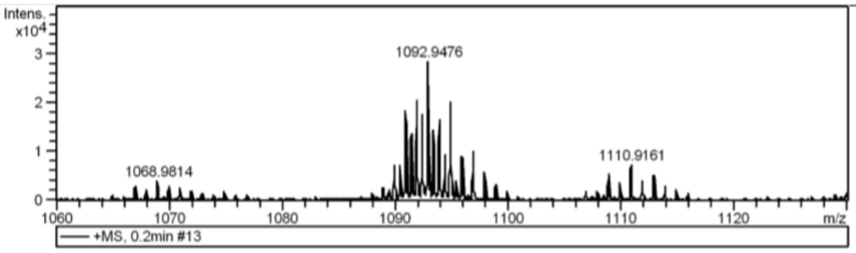

#	m/z	Res.	S/N	I	FWHM
1	1066.9869	9400	9.7	2834	0.1135
2	1067.9817	7952	7.4	1968	0.1343
3	1068.9814	10981	14.6	3906	0.0973
4	1069.9786	10785	9.9	2644	0.0992
5	1070.9706	9210	8.4	2252	0.1163
6	1071.9658	9511	5.9	1590	0.1127
7	1072.9369	6776	5.3	1418	0.1583
8	1089.9571	8824	25.9	7042	0.1235
9	1090.9504	11116	66.1	18001	0.0981
10	1091.9497	10144	74.9	20398	0.1076
11	1092.9476	11204	103.9	28342	0.0975
12	1093.9480	9776	60.1	16408	0.1119
13	1094.9489	11109	72.9	19923	0.0986
14	1095.9458	9509	32.0	8743	0.1153
15	1096.9488	10025	36.1	9878	0.1094
16	1097.9485	11254	20.6	5637	0.0976
17	1098.9477	9749	11.6	3171	0.1127
18	1099.9481	13973	6.5	1780	0.0787
19	1108.9187	10508	19.1	5274	0.1055
20	1109.9215	11613	12.6	3491	0.0956
21	1110.9161	10375	25.9	7177	0.1071
22	1111.9170	9815	13.7	3785	0.1133
23	1112.9172	10327	17.6	4877	0.1078
24	1113.9176	12229	10.3	2869	0.0911
25	1114.9106	10046	6.8	1898	0.1110
26	1115.9156	27048	4.3	1196	0.0413

Supplementary Figure 12. HRMS spectrum of compound **4**.

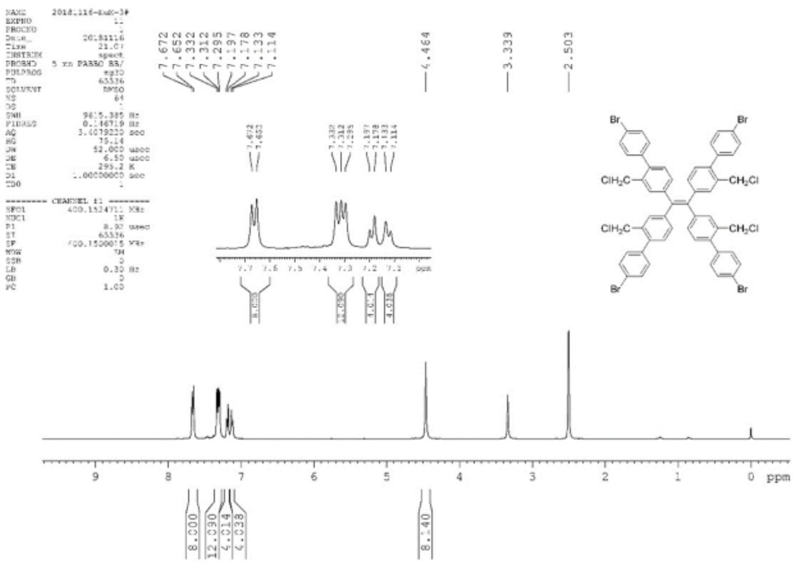

Supplementary Figure 13. ^{1}H NMR spectrum of compound **5** in DMSO-d_6.

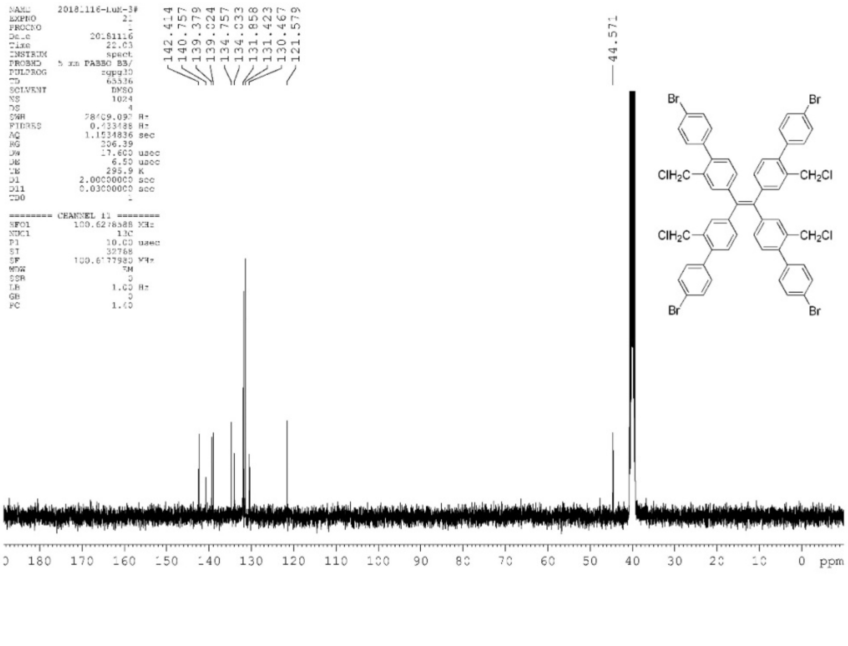

Supplementary Figure 14. ^{13}C NMR spectrum of compound **5** in DMSO-d_6.

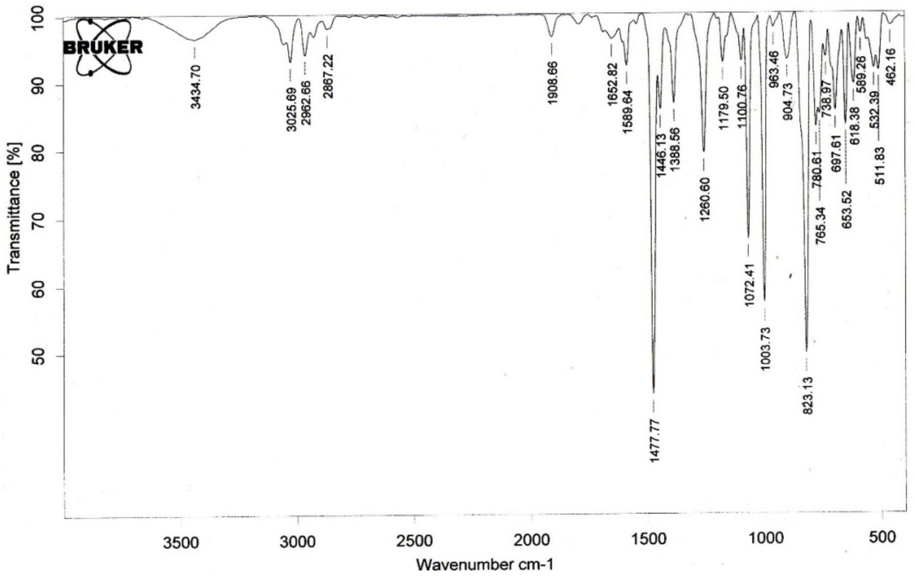

Supplementary Figure 15. IR spectrum of compound 5.

Mass Spectrum List Report

Analysis Info

Analysis Name D:\Data\ZhengYS\ZHENG-hum20190425-3-2.d
Method tune_wide.m
Sample Name ZHENG-hum20190425-3-2
Comment

Acquisition Date 4/25/2019 11:07:21 AM
Operator BDAL@DE
Instrument / Ser# micrOTOF 10401

Acquisition Parameter

Source Type	ESI	Ion Polarity	Positive	Set Nebulizer	0.3 Bar
Focus	Active			Set Dry Heater	180 °C
Scan Begin	50 m/z	Set Capillary	4500 V	Set Dry Gas	4.0 l/min
Scan End	3000 m/z	Set End Plate Offset	-500 V	Set Divert Valve	Waste

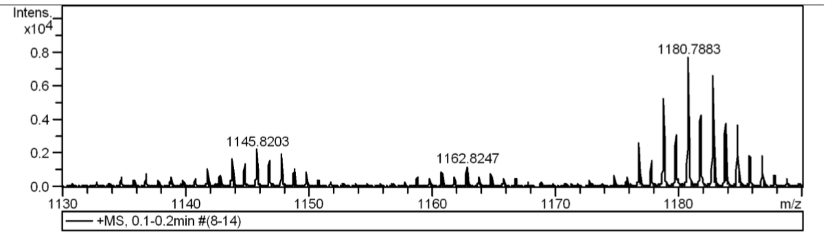

#	m/z	Res.	S/N	I	FWHM
1	1143.8226	9838	15.9	1703	0.1163
2	1144.8269	11738	12.8	1306	0.0975
3	1145.8203	11632	21.1	2147	0.0985
4	1146.8208	13147	14.4	1459	0.0872
5	1147.8201	13269	18.4	1864	0.0865
6	1162.8247	13731	11.5	1127	0.0847
7	1176.7926	13723	26.7	2548	0.0858
8	1178.7935	11124	54.3	5169	0.1060
9	1179.7932	11381	32.6	3095	0.1037
10	1180.7883	11818	80.5	7625	0.0999
11	1181.7914	12534	44.5	4206	0.0943
12	1182.7872	12292	69.5	6561	0.0962
13	1183.7874	13184	39.7	3742	0.0898
14	1184.7832	10720	38.8	3648	0.1105

Supplementary Figure 16. HRMS spectrum of compound **5**.

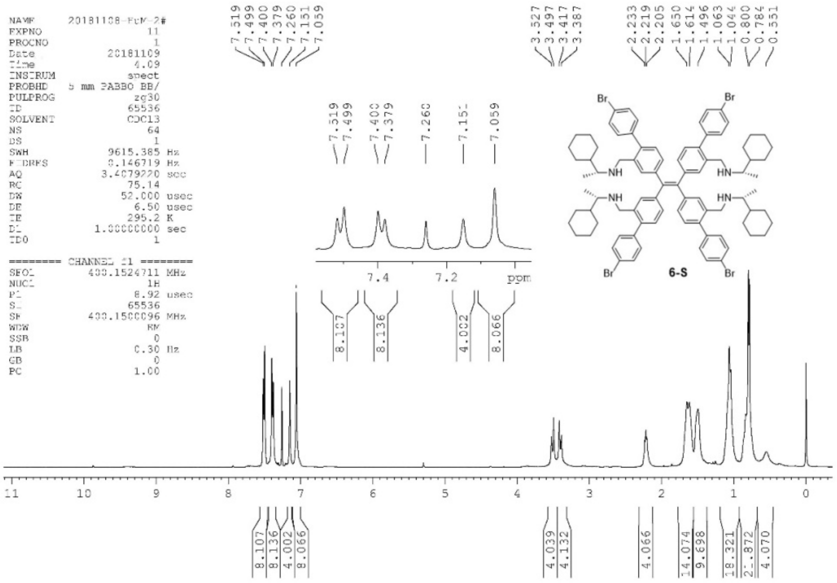

Supplementary Figure 17. ^{1}H NMR spectrum of compound (*S*)-**6** in $CDCl_3$.

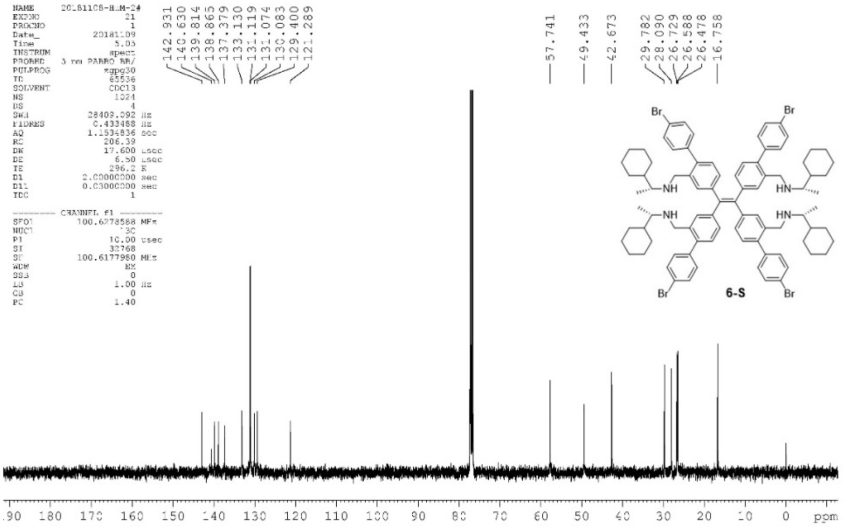

Supplementary Figure 18. ^{13}C NMR spectrum of compound (*S*)-**6** in $CDCl_3$.

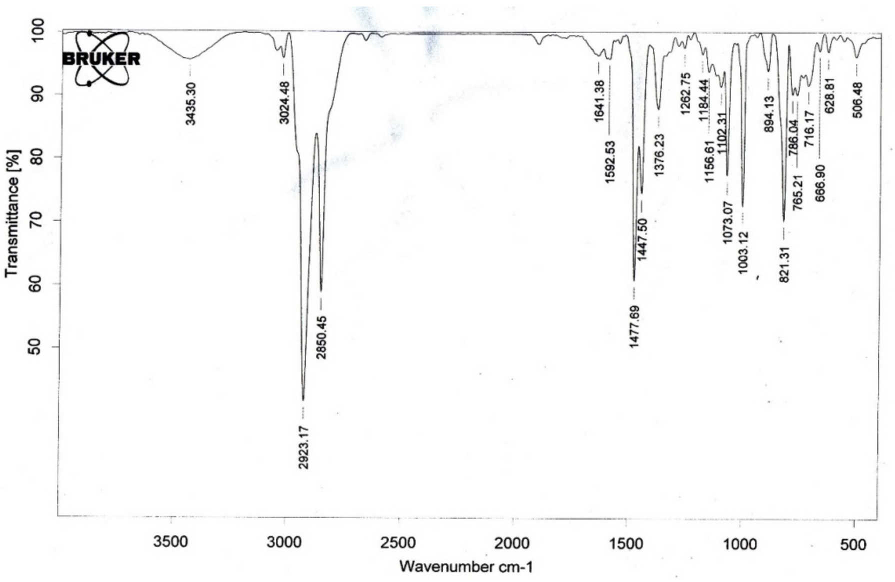

Supplementary Figure 19. IR spectrum of compound (*S*)-**6**.

Mass Spectrum List Report

Analysis Info

Analysis Name	E:\ºúÃ÷\ÖÊÆ×\zheng-huming20190404-2.d	Acquisition Date	2019/4/4 13:12:58
Method	tune_wide.m	Operator	BDAL@DE
Sample Name	zheng-huming20190404-2	Instrument / Ser#	micrOTOF 10401
Comment			

Acquisition Parameter

Source Type	ESI	Ion Polarity	Positive	Set Nebulizer	0.3 Bar
Focus	Active			Set Dry Heater	180 °C
Scan Begin	50 m/z	Set Capillary	4500 V	Set Dry Gas	4.0 l/min
Scan End	3000 m/z	Set End Plate Offset	-500 V	Set Divert Valve	Waste

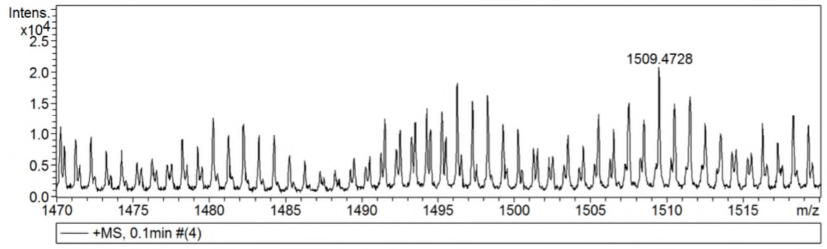

#	m/z	Res.	S/N	I	FWHM
1	1505.4953	16072	5.7	10315	0.0937
2	1506.2648	18684	1.7	3157	0.0806
3	1506.4966	14605	4.3	7798	0.1031
4	1507.4829	11331	6.6	12042	0.1330
5	1508.4731	12877	5.1	9321	0.1171
6	1509.4728	15304	9.7	20741	0.0986
7	1510.4741	12973	6.5	12026	0.1164
8	1511.4835	11153	7.1	13099	0.1355
9	1512.4847	11495	4.7	8732	0.1316
10	1513.4936	9853	3.8	7038	0.1536
11	1545.4488	14588	33.1	60553	0.1059
12	1571.4246	15167	28.4	48266	0.1036

Supplementary Figure 20. HRMS spectrum of compound (*S*)-**6**.

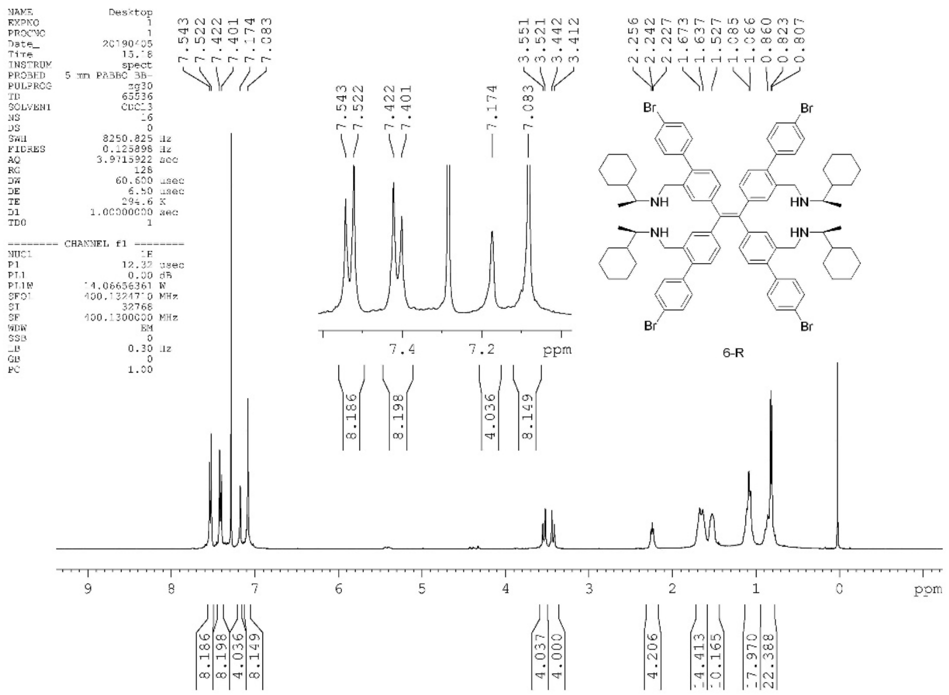

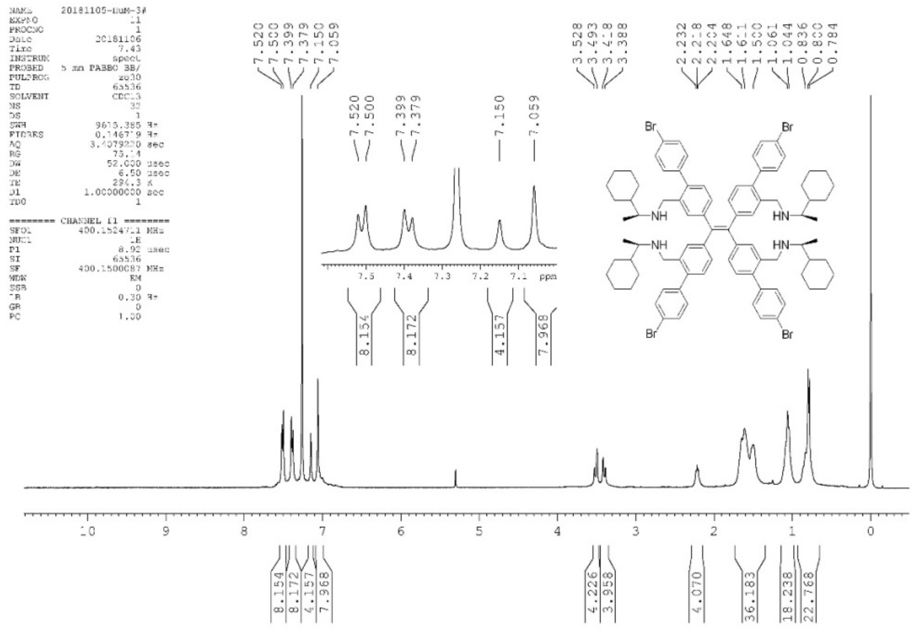

Supplementary Figure 21. ^{1}H NMR spectrum of compound (*R*)-**6** in $CDCl_3$.

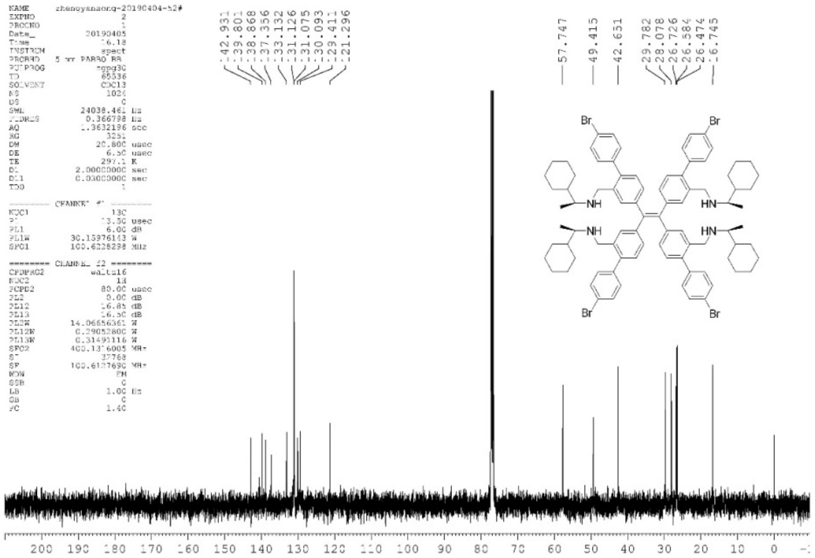

Supplementary Figure 22. ^{13}C NMR spectrum of compound (*R*)-**6** in $CDCl_3$.

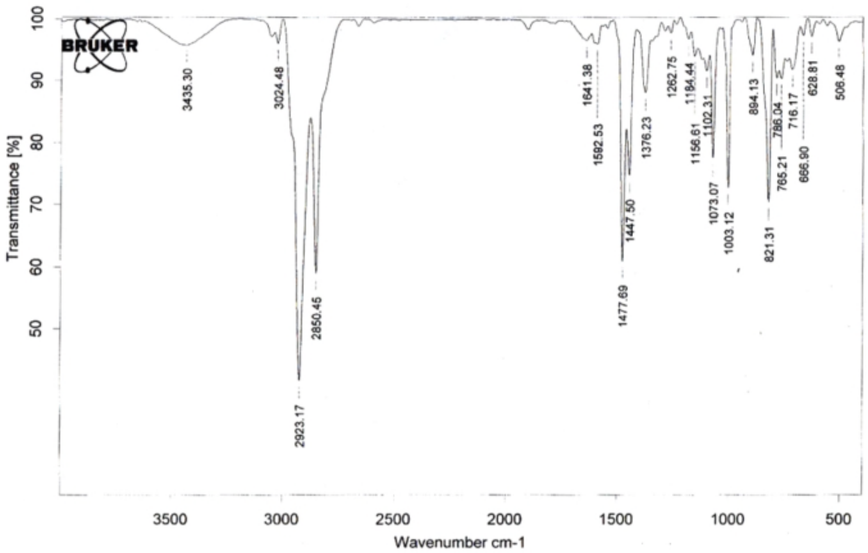

Supplementary Figure 23. IR spectrum of compound (*R*)-**6**.

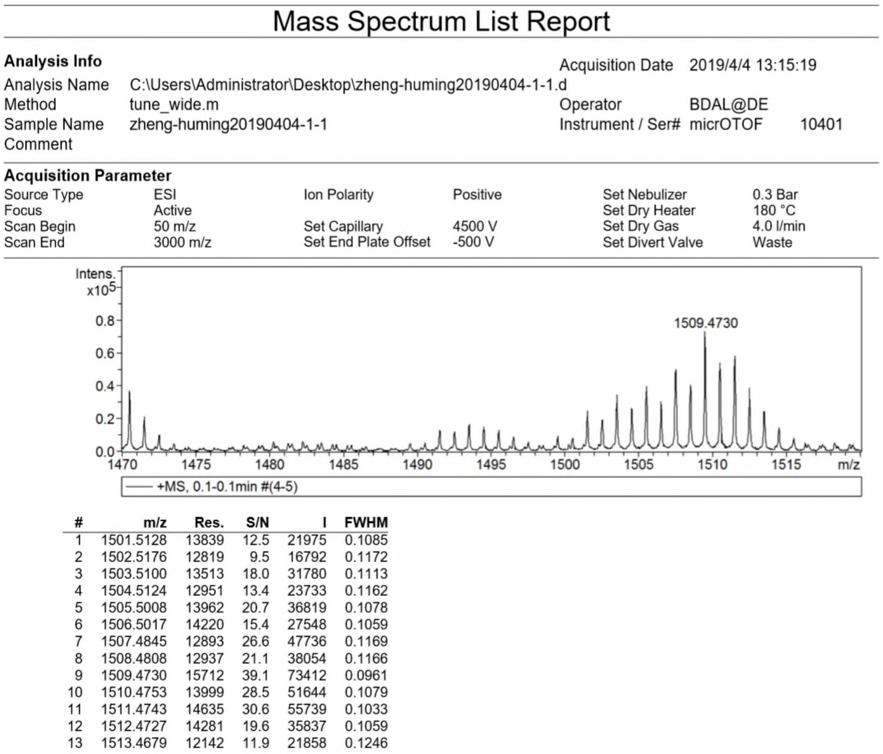

Mass Spectrum List Report

Analysis Info

Analysis Name	C:\Users\Administrator\Desktop\zheng-huming20190404-1-1.d	Acquisition Date	2019/4/4 13:15:19
Method	tune_wide.m	Operator	BDAL@DE
Sample Name	zheng-huming20190404-1-1	Instrument / Ser#	micrOTOF 10401
Comment			

Acquisition Parameter

Source Type	ESI	Ion Polarity	Positive	Set Nebulizer	0.3 Bar
Focus	Active			Set Dry Heater	180 °C
Scan Begin	50 m/z	Set Capillary	4500 V	Set Dry Gas	4.0 l/min
Scan End	3000 m/z	Set End Plate Offset	-500 V	Set Divert Valve	Waste

#	m/z	Res.	S/N	I	FWHM
1	1501.5128	13839	12.5	21975	0.1085
2	1502.5176	12819	9.5	16792	0.1172
3	1503.5100	13513	18.0	31780	0.1113
4	1504.5124	12951	13.4	23733	0.1162
5	1505.5008	13962	20.7	36819	0.1078
6	1506.5017	14220	15.4	27548	0.1059
7	1507.4845	12893	26.6	47736	0.1169
8	1508.4808	12937	21.1	38054	0.1166
9	1509.4730	15712	39.1	73412	0.0961
10	1510.4753	13999	28.5	51644	0.1079
11	1511.4743	14635	30.6	55739	0.1033
12	1512.4727	14281	19.6	35837	0.1059
13	1513.4679	12142	11.9	21858	0.1246

Supplementary Figure 24. HRMS spectrum of compound (*R*)-**6**.

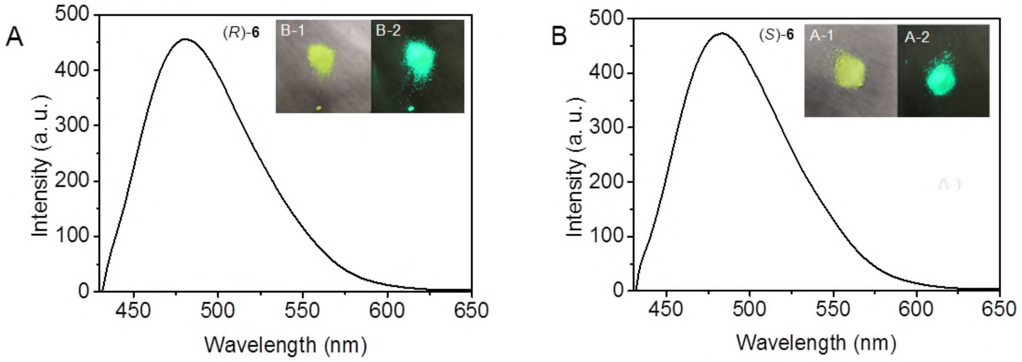

Supplementary Figure 25. Emission spectra of (*R*)-**6** (A) and (*S*)-**6** (B) as solid powder. Inset, photos of solid powder of (*R*)-**6** (A) and (*S*)-**6** under daylight (B-1 and A-1) and under a portable 365 nm lamp (B-2 and A-2). λ_{ex} = 405 nm, ex/em slit widths = 10/10 nm.

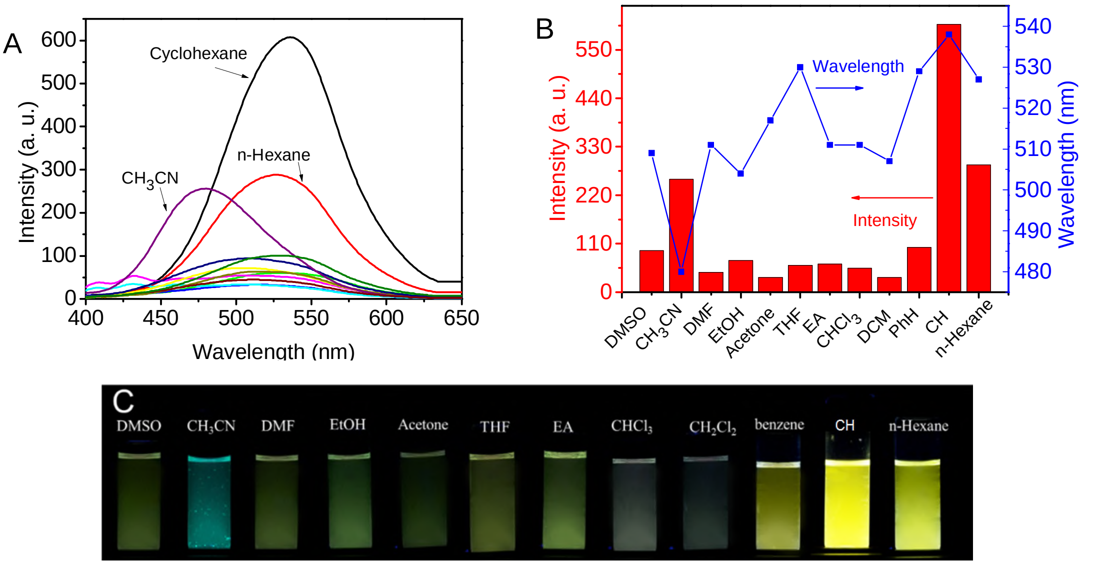

Supplementary Figure 26. (A) Emission spectra of (S)-**6** in different solvents. (B) Change in intensity and emission maximum wavelength of emission spectrum of (S)-**6** with different solvents. (C) Photos of (S)-**6** in different solvents under a portable 365 nm lamp. [(S)-**6**] = 2.0 × 10^{-5} M, λ_{ex} = 363 nm, ex/em slit widths = 3/3 nm; EtOH: ethanol; EA: Ethyl acetate, CH: cyclohexane; the solution of (S)-**6** was homogeneous and clear in all solvents except acetonitrile in which precipitates appeared.

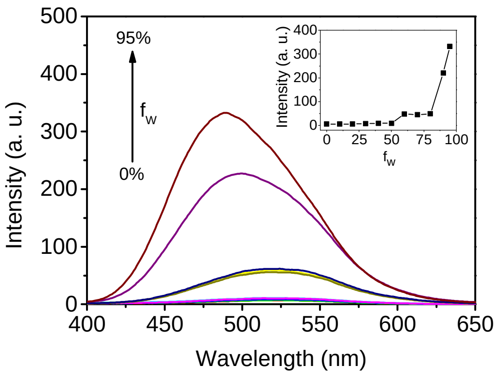

Supplementary Figure 27. Emission spectra of (S)-**6** in a mixed solvent of water/THF with water fractions (f_w, volume percent, same below). Inset, curve of fluorescent intensity vs. water fraction. λ_{ex} = 363 nm, ex/em slit widths = 1.5/3 nm, [(S)-**6**] = 1.0 × 10^{-5} M).

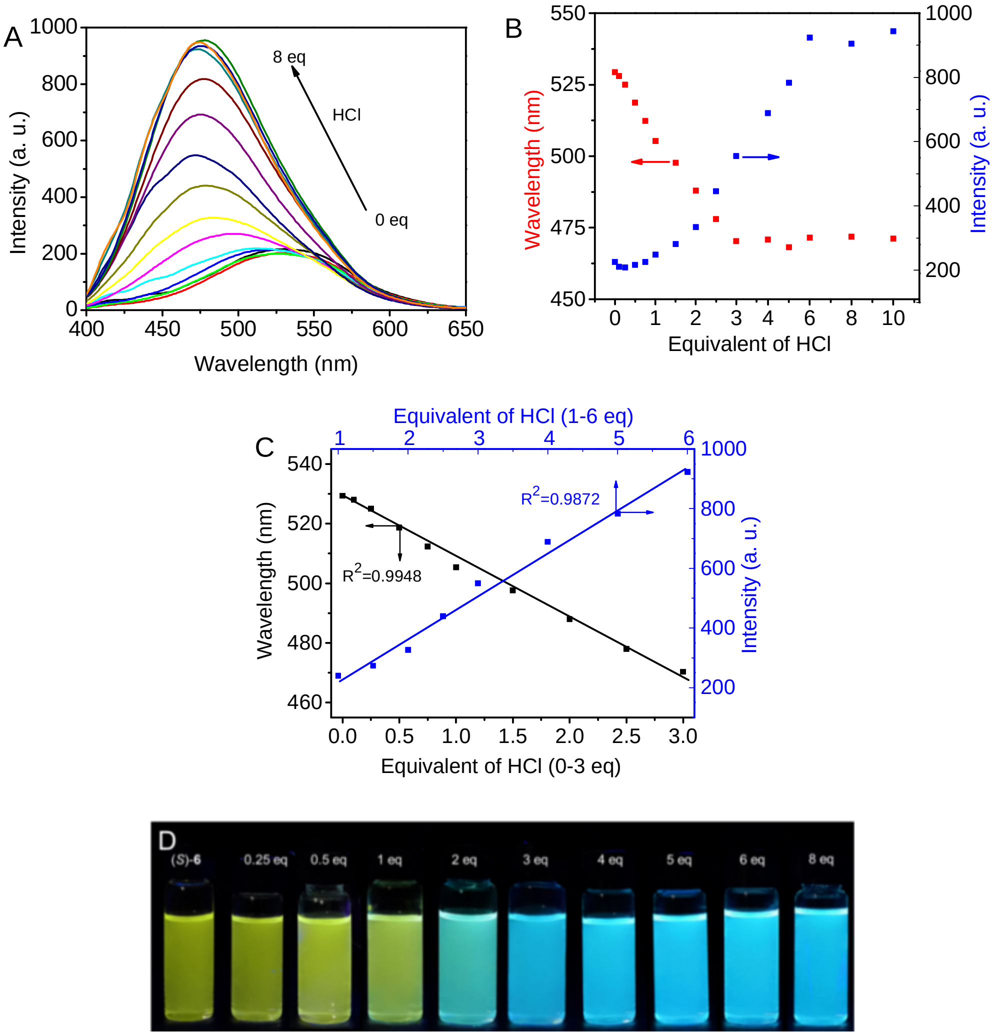

Supplementary Figure 28. (A) Change in emission spectra of (*S*)-**6** in THF with molar equivalent of added hydrochloric acid. (B) Change of emission maximum wavelength and emission intensity with molar equivalent of added hydrochloric acid. (C) Line graph of molar equivalent of hydrochloric acid versus emission wavelength and emission intensity of (*S*)-**6** in THF, respectively. (D) Photos of solution of (*S*)-**6** in THF with molar equivalent of hydrochloric acid. [(*S*)-**6**] = 2.0 × 10^{-5} M, λ_{ex} = 363 nm, ex/em slit widths = 5/3 nm.

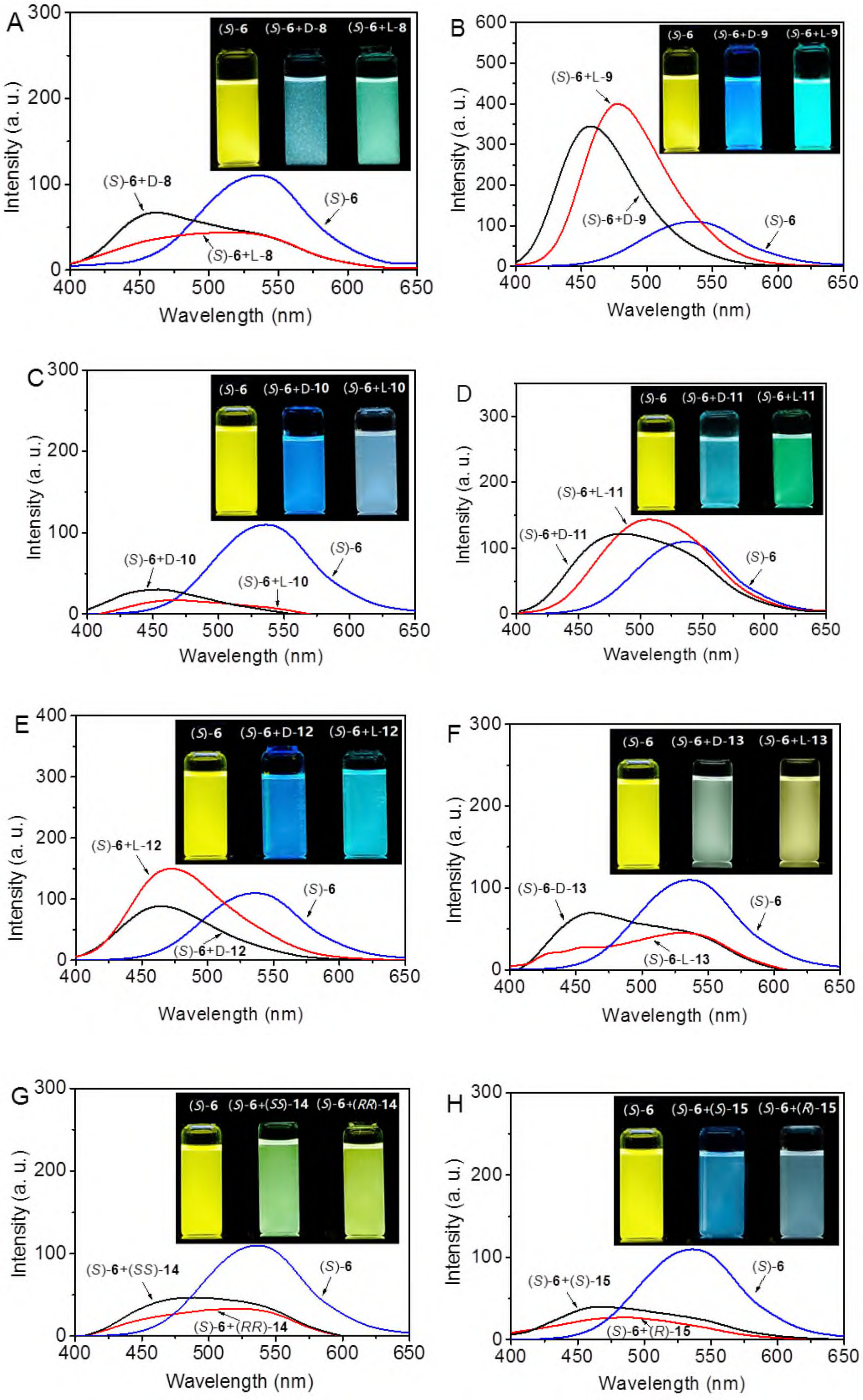
A
(S)-6
(S)-6+D-8
(S)-6+L-8
Intensity (a. u.)
Wavelength (nm)
B
(S)-6
(S)-6+D-9
(S)-6+L-9
Intensity (a. u.)
Wavelength (nm)
C
(S)-6
(S)-6+D-10
(S)-6+L-10
Intensity (a. u.)
Wavelength (nm)
D
(S)-6
(S)-6+D-11
(S)-6+L-11
Intensity (a. u.)
Wavelength (nm)
E
(S)-6
(S)-6+D-12
(S)-6+L-12
Intensity (a. u.)
Wavelength (nm)
F
(S)-6
(S)-6+D-13
(S)-6+L-13
(S)-6-D-13
(S)-6-L-13
Intensity (a. u.)
Wavelength (nm)
G
(S)-6
(S)-6+(SS)-14
(S)-6+(RR)-14
Intensity (a. u.)
Wavelength (nm)
H
(S)-6
(S)-6+(S)-15
(S)-6+(R)-15
Intensity (a. u.)
Wavelength (nm)

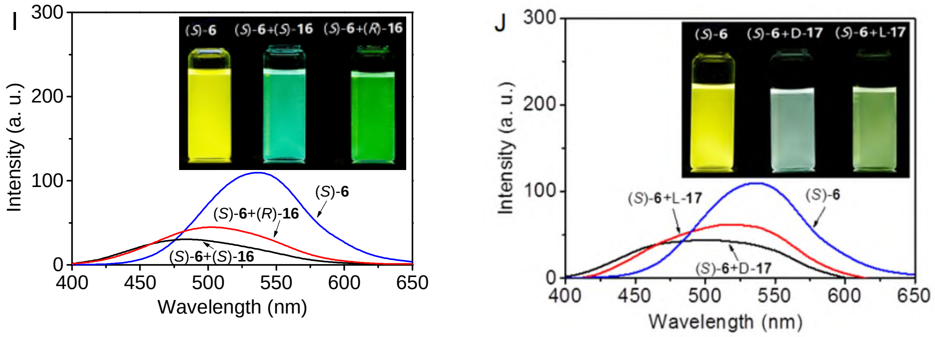

Supplementary Figure 29. Emission spectra of (*S*)-**6** after respectively mixed with two enantiomers of a variety of chiral acids in the mixed solvent of cyclohexane/acetone 98:2. (A) Di-*p*-anisoyltartaric acid **8**/(*S*)-**6** 1:1, (B) Dibenzoyltartaric acid **9**/(*S*)-**6** 2:1. (C) Tartaric acid **10**/(*S*)-**6** 2:1. (D) Boc-glutamic acid **11**/(*S*)-**6** 2:1. (E) Boc-aspartic acid **12**/(*S*)-**6** 3:1. (F) Malic acid **13**/(*S*)-**6** 2:1. (G) 1,1-Cyclohexanedicarboxylic acid **14**/(*S*)-**6** 4:1. (H) Mandelic acid **15**/(*S*)-**6** 3:1. (I) 2-Chloromandelic acid **16**/(*S*)-**6** 4:1. (J) Pyroglutamic acid **17**/(*S*)-**6** 4:1. Insets, photos of solution of (*S*)-**6** without and with chiral acid enantiomers under irradiation of 365 nm light. [(*S*)-**6**] = 1.0 × 10^{-5} M.

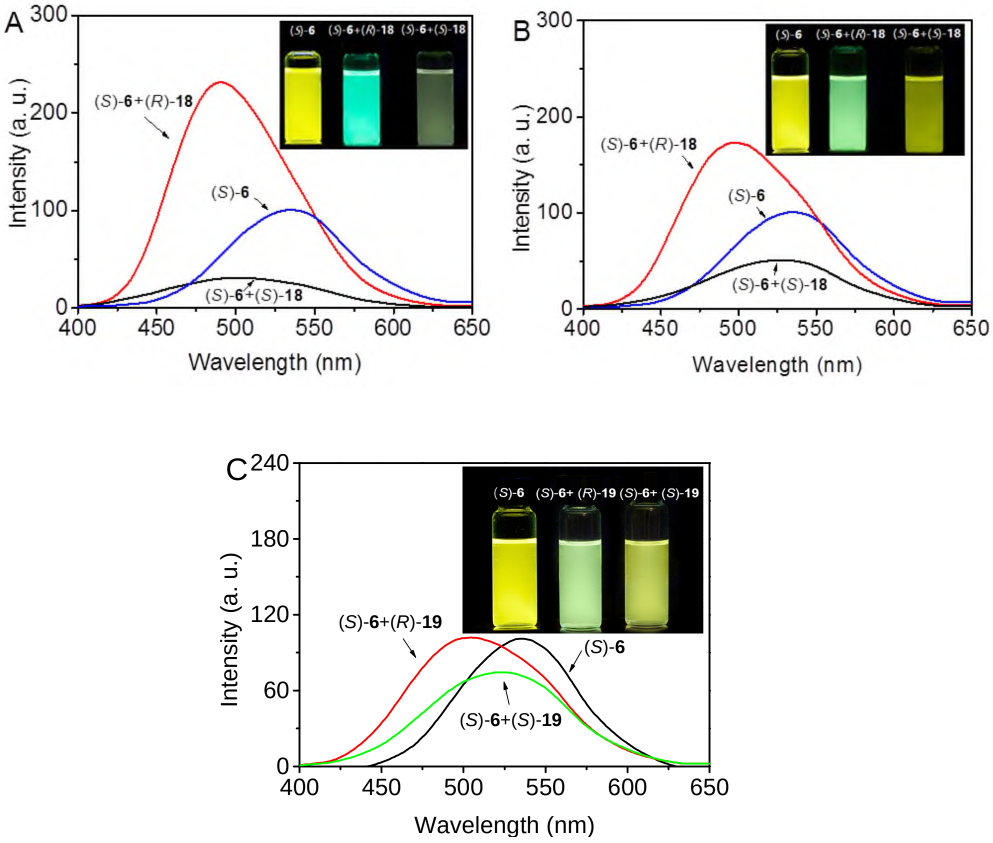

Supplementary Figure 30. Emission spectra of (*S*)-**6** after respectively mixed with two enantiomers of 2-(2,4-dichlorophenoxy)propionic acid **18** (herbicide) with (A) **18**/(*S*)-**6** 4:1 and (B) **18**/(*S*)-**6** 2:1. (C) Emission spectra of (*S*)-**6** after respectively mixed with two enantiomers of 2-(4-chloro-2-methylphenoxy)propionic acid **19** with **19**/(*S*)-**6** 4:1. Insets, photos of solution of (*S*)-**6** without and with chiral acid enantiomers under irradiation of 365 nm light. [(*S*)-**6**] = 1.0 × 10^{-5} M in cyclohexane/acetone 98:2.

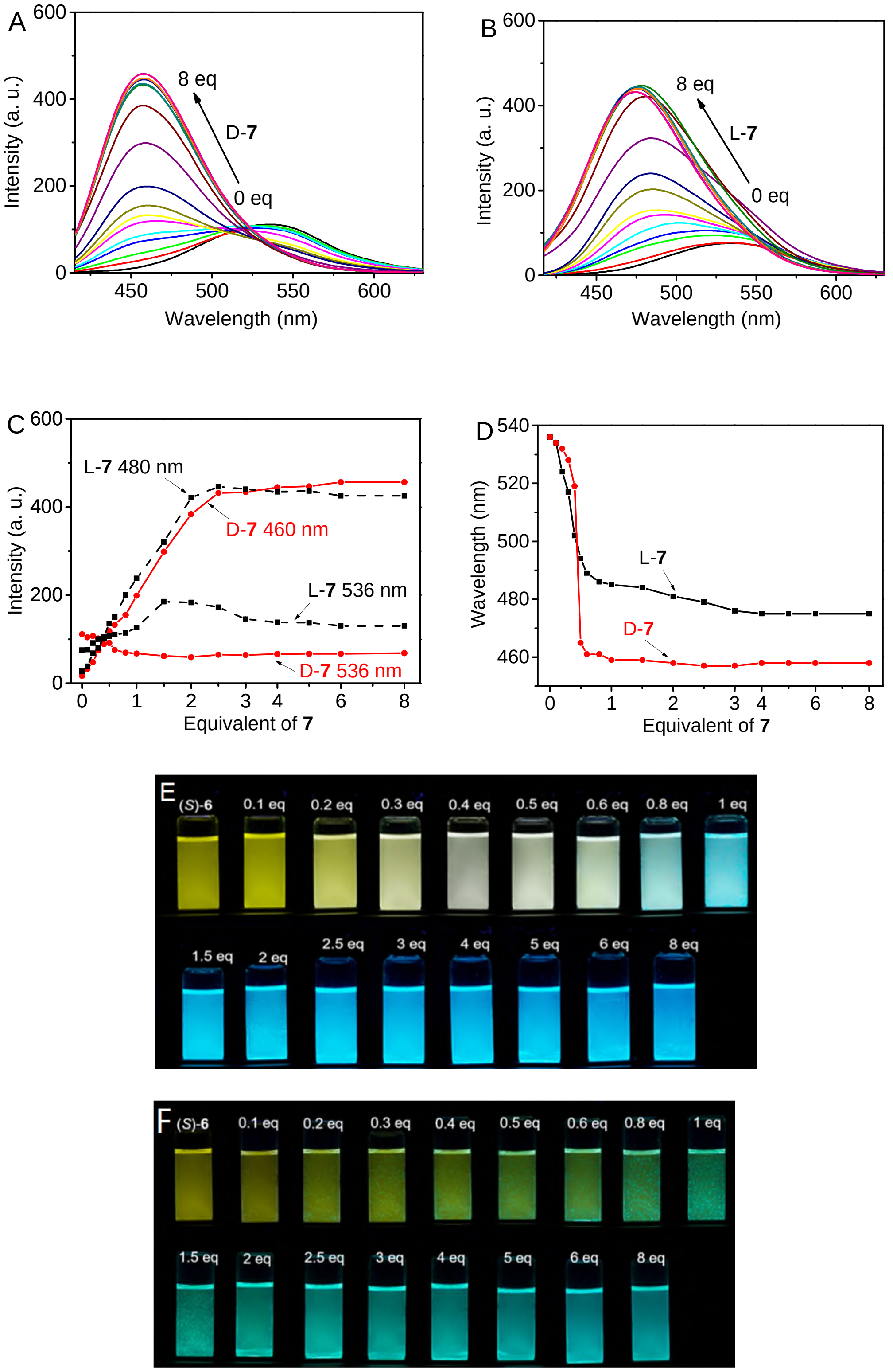

Supplementary Figure 31. Change in emission spectra of (*S*)-**6** with molar equivalent of added D-**7** (A) and L-**7** (B). (C) Change of emission intensity with D-**7** and L-**7**. (D) Change of the emission λ_{max} with D-**7** and L-**7**. Photos of solution of (*S*)-**6** in the presence of D-**7** (E) and L-**7** (F) under 365 nm light. [(*S*)-**6**] = 1.0 × 10^{-5} M in cyclohexane/acetone 98:2, λ_{ex} = 363 nm, ex/em slit widths = 1.5/3 nm.

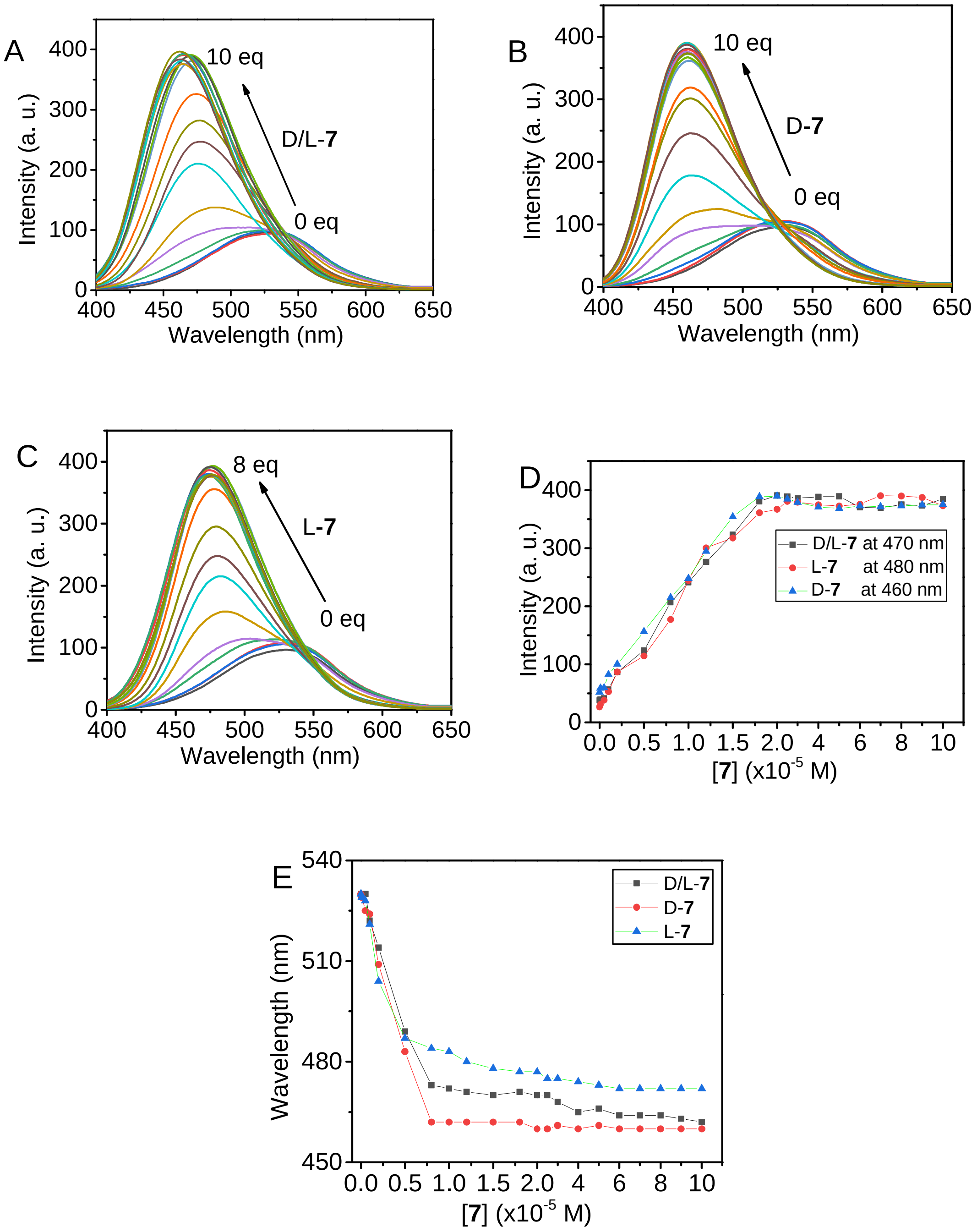

Supplementary Figure 32. Change in emission spectra of (*S*)-**6** with concentration of added D-**7** (A), L-**7** (B), and racemic **7** (D/L-**7**) (C). (D) Change of emission intensity with concentration of D-**7**, L-**7** and D/L-**7**. (E) Change of the emission λ_{max} with concentration of D-**7**, L-**7** and D/L-**7**. [(*S*)-**6**] = 1.0×10^{-5} M in cyclohexane/acetone 98:2, λ_{ex} = 363 nm, ex/em slit widths = 1.5/3 nm.

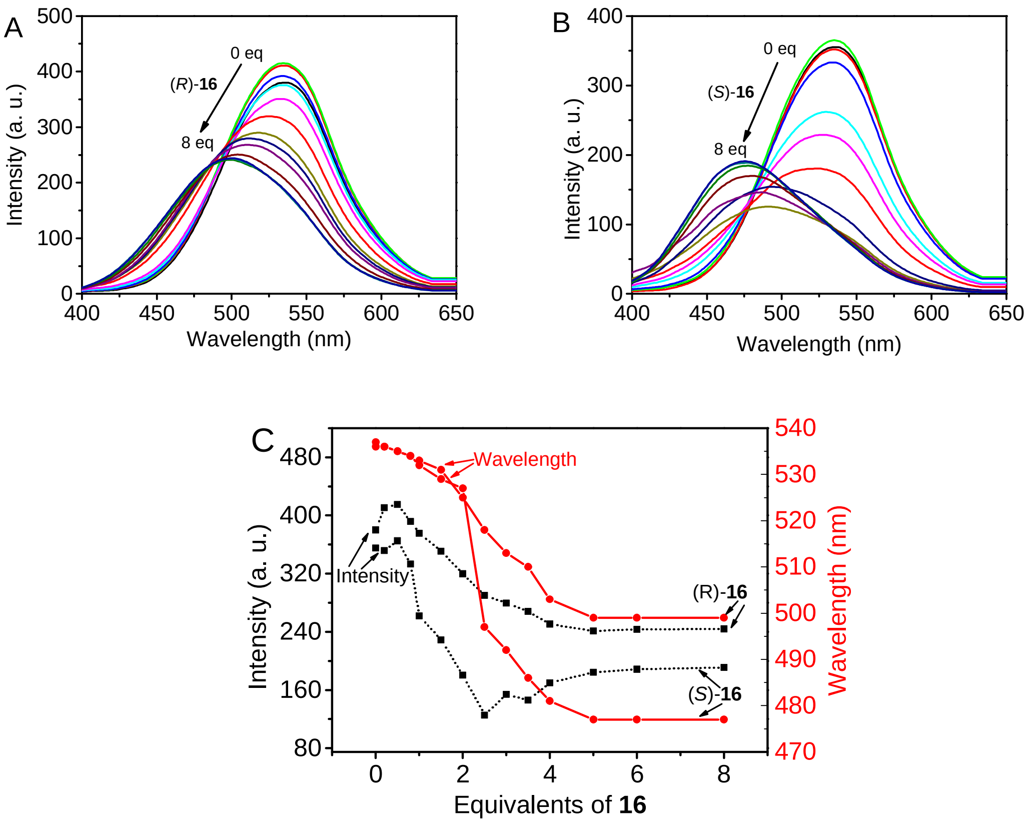

Supplementary Figure 33. Change in emission spectra of (*S*)-**6** with molar equivalents of (*R*)-**16** (A) or **(***S***)-16** (B). (C) Change of emission maximum wavelength and emission intensity with molar equivalents of (*R*)-**16** or **(***S***)-16**. [(*S*)-**6**] = 1.0 × 10^{-5} M in cyclohexane/acetone 98:2, λ_{ex} = 363 nm, ex/em slit widths = 3/3 nm.

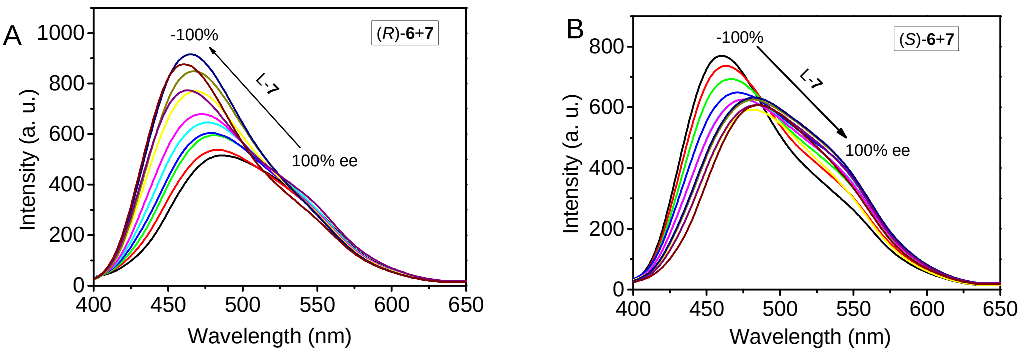

Supplementary Figure 34 (A) Emission spectrum of the solution of (*R*)-**6** was changed with ee% of L-**7**. (B) Emission spectrum of the solution of (*S*)-**6** was changed with ee% of L-**7**. [(*R*)-**6**] = [(*S*)-**6**] = = [**7**] = 1.0 × 10^{-5} M in cyclohexane/acetone 98:2.

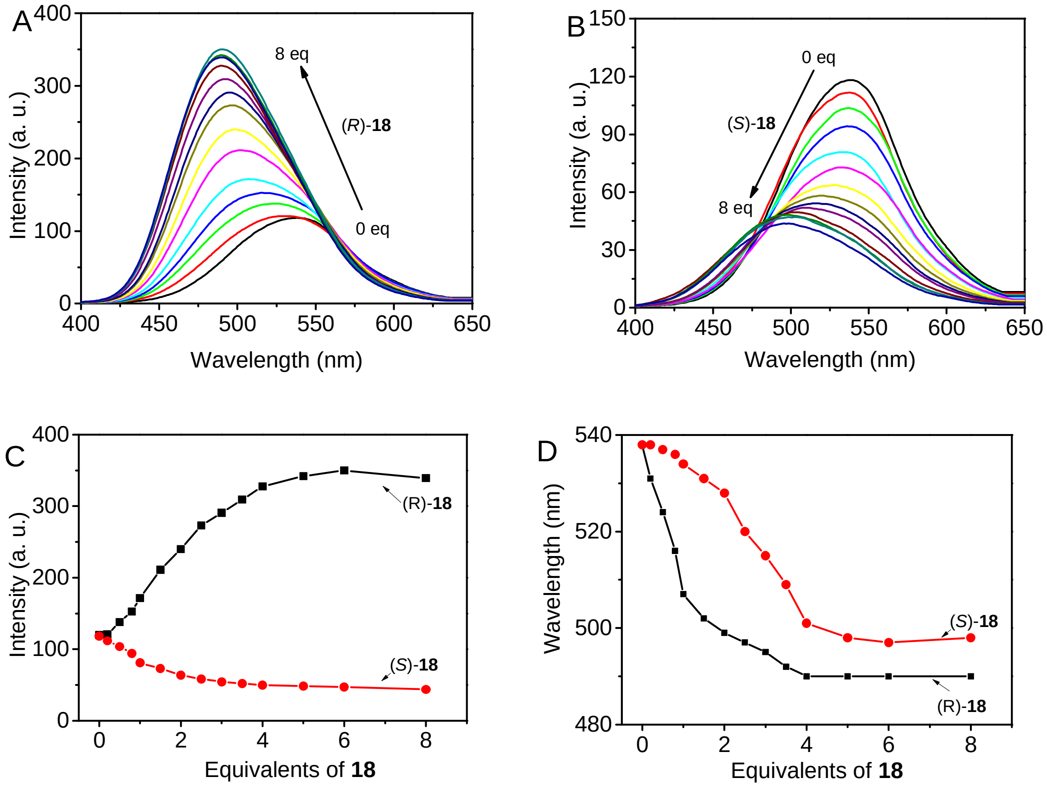

Supplementary Figure 35. Change in emission spectra of (*S*)-**6** with molar equivalents of (*R*)-**18** (A) or (*S*)-**18** (B). And change of emission intensity (C) and emission maximum wavelength (D) with molar equivalents of (*R*)-**18** or (*S*)-**18**. [(*S*)-**6**] = 1.0 × 10^{-5} M in cyclohexane/acetone 98:2, λ_{ex} = 363 nm, ex/em slit widths = 1.5/3 nm.

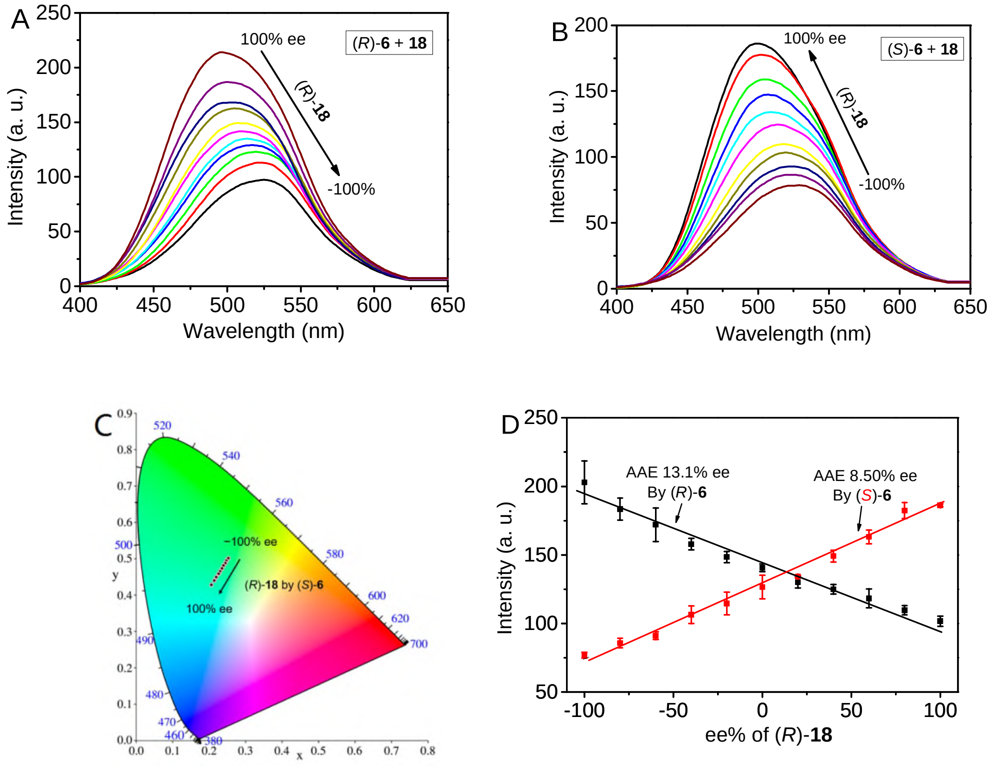

Supplementary Figure 36. (A) Emission spectrum of the solution of (*R*)-**6** was changed with ee% of (*R*)-**18**. (B) Emission spectrum of the solution of (*S*)-**6** was changed with ee% of (*R*)-**18**. (C) CIE chromaticity diagram of ee% of (*R*)-**18** measured by (*R*)-**6**. (D) Change of emission intensity of **6** with ee% of (*R*)-**18**. [(*R*)-**6**] = [(*S*)-**6**] = 1/2[**18**] = 1.0 × 10^{-5} M in cyclohexane/acetone 98:2; λ_{ex} = 363 nm, ex/em slit widths = 1.5/3 nm.

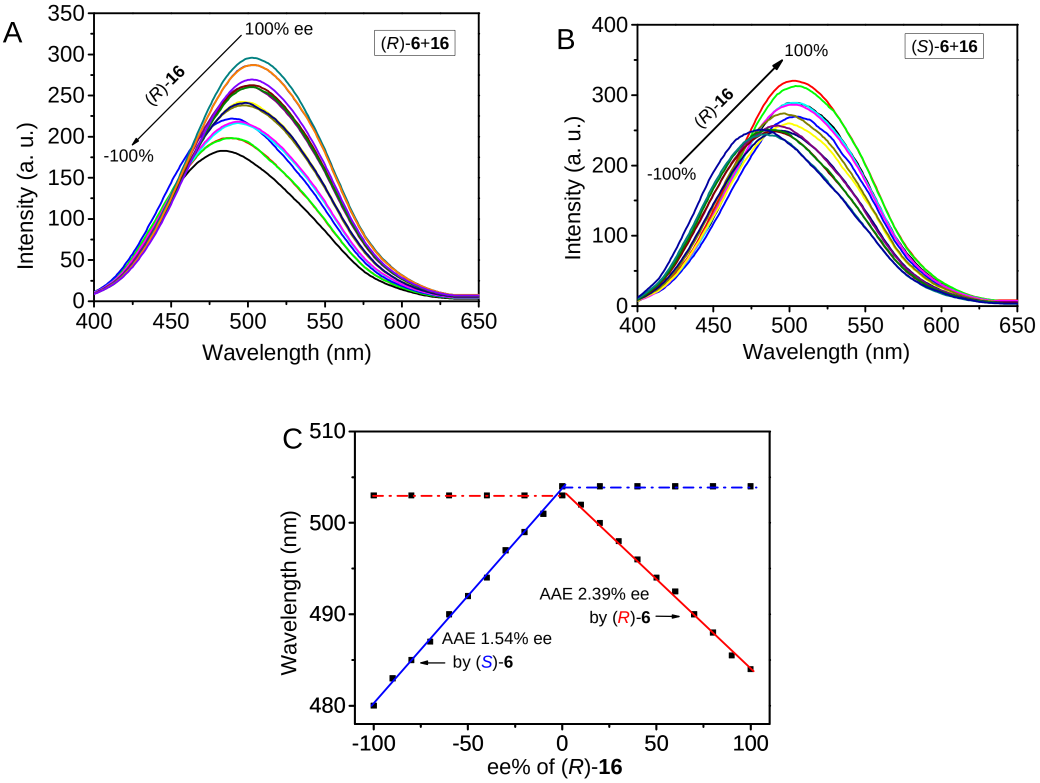

Supplementary Figure 37. (A) Emission spectrum of the solution of (*R*)-**6** was changed with ee% of (*R*)-**16**. (B) Emission spectrum of the solution of (*S*)-**6** was changed with ee% of (*R*)-**16**. (C) Change of emission maximum wavelength of **6** with ee% of (*R*)-**16**. [(*R*)-**6**] = [(*S*)-**6**] = 1/4[**16**] = 1.0 × 10^{-5} M in cyclohexane/acetone 98:2; λ_{ex} = 363 nm, ex/em slit widths = 3/3 nm.

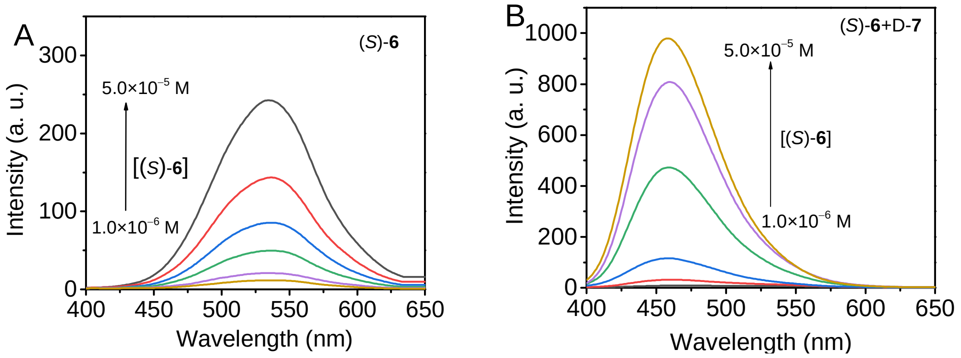

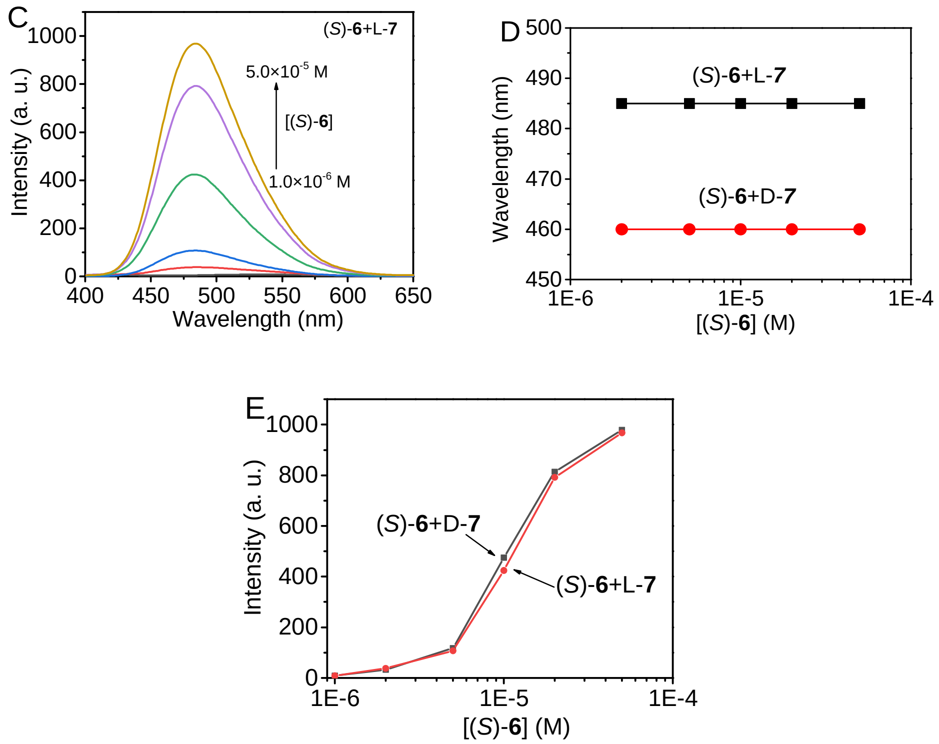

Supplementary Figure 38. Change in emission spectra of (*S*)-**6** (A) and a mixture (1:1) of (*S*)-**6** and D-7 (B) or a mixture (1:1) of (*S*)-**6** and L-7 (C) with concentration in hexane/acetone 98:2. And change of emission wavelength (D) and emission intensity (E) of (*S*)-**6** with concentration of the mixture of it and one enantiomer of **7**. λ_{ex} = 363 nm, em/ex slit widths = 1.5/3 nm.

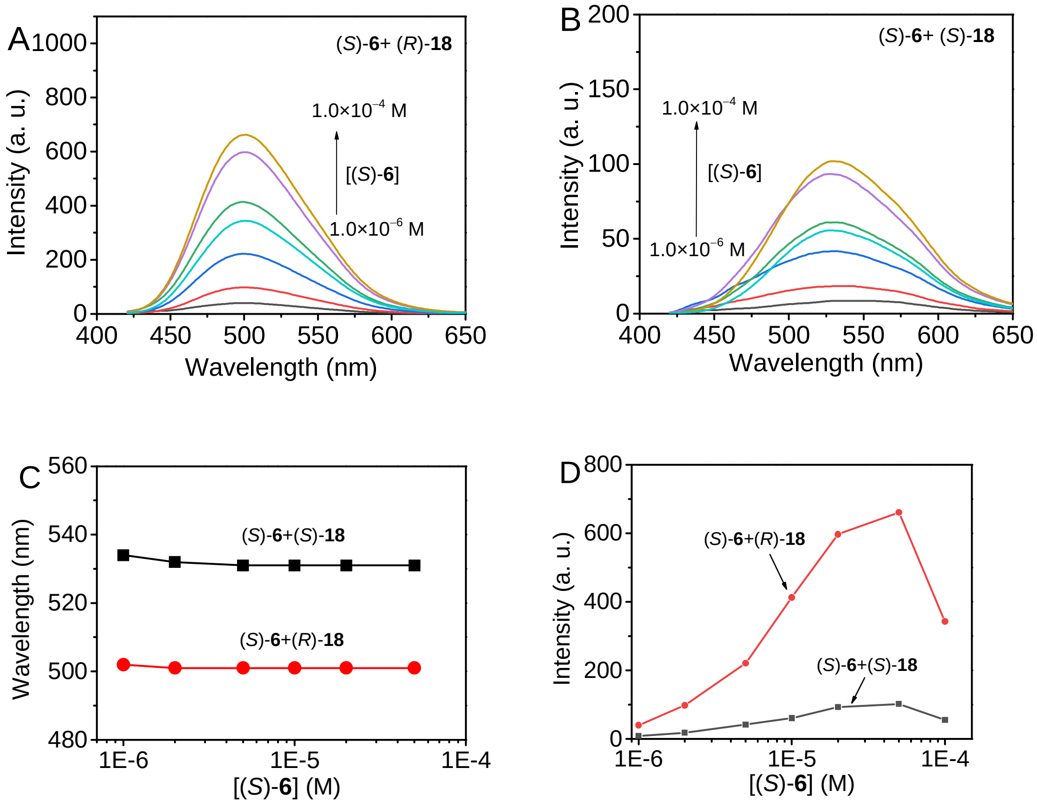

Supplementary Figure 39. Change in emission spectra of a mixture (1:2) of (*S*)-**6** and (*R*)-**18** (A) or a mixture (1:4) of (*S*)-**6** and (*S*)-**18** (B) with concentration in cyclohexane/acetone 98:2. And change of emission wavelength (C) and emission intensity (D) of (*S*)-**6** with concentration of the mixture of it and one enantiomer of **18**. λ_{ex} = 363 nm, em/ex slit widths = 1.5/3 nm.

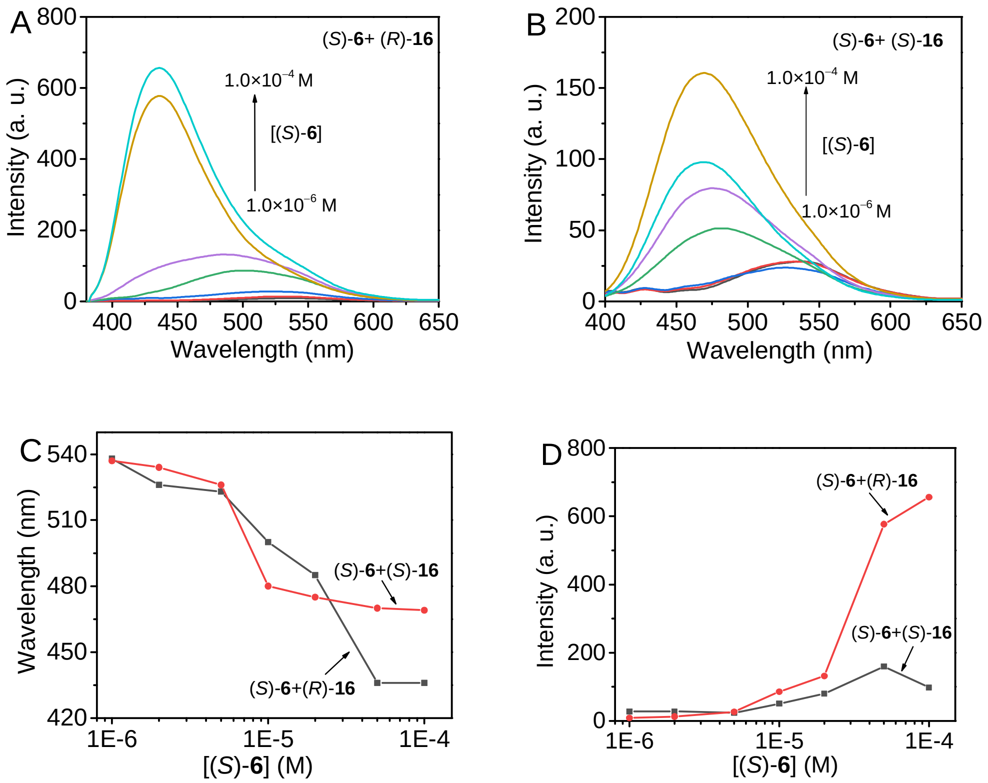

Supplementary Figure 40. Change in emission spectra of a mixture (1:4) of (*S*)-**6** and (*R*)-**16** (A) or a mixture (1:4) of (*S*)-**6** and (*S*)-**16** (B) with concentration in cyclohexane/acetone 98:2. And change of emission wavelength (C) and emission intensity (D) of (*S*)-**6** with concentration of the mixture of it and one enantiomer of **16**. λ_{ex} = 363 nm, em/ex slit widths = 1.5/3 nm.

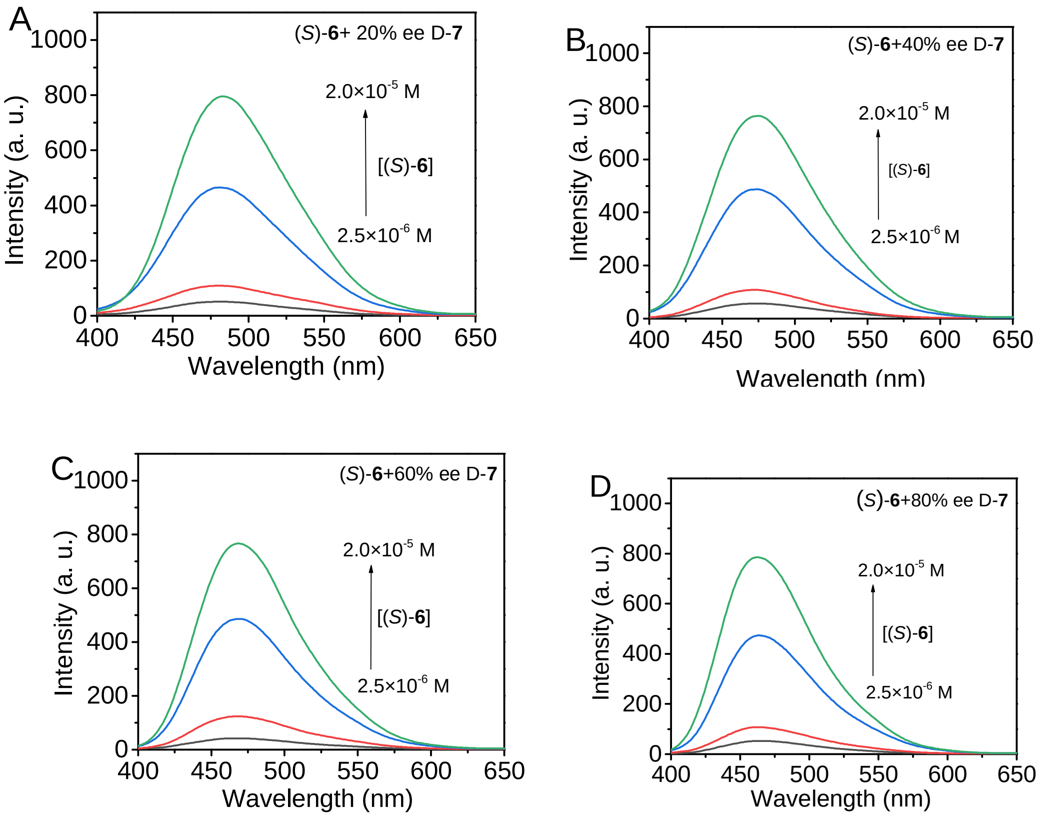

Supplementary Figure 41. Change in emission spectra of (*S*)-**6** with concentration of it in the presence of 20% ee D-**7** (A), 40% ee D-**7** (B), 60% ee D-**7** (C), and 80% ee D-**7** (D) in cyclohexane/acetone 98:2. [(S)-**6**]/[**7**] = 1:2; [(*S*)-**6**] was changed from 2.5 × 10^{-6} M, 5.0 × 10^{-6} M, 1.0 × 10^{-5} M, to 2.0 × 10^{-5} M. λ_{ex} = 363 nm, em/ex slit widths = 1.5/3 nm.

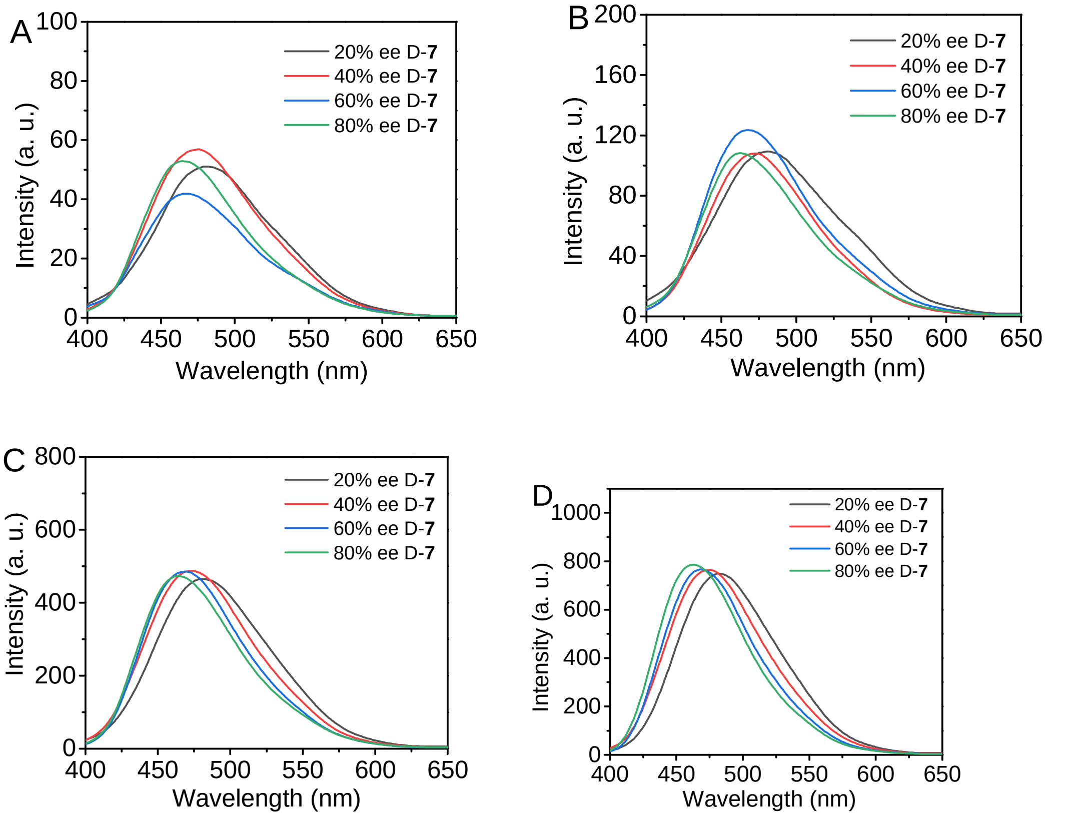

Supplementary Figure 42. Change in emission spectra of (*S*)-**6** with enantiomeric composition of **7** when concentration of (*S*)-**6** was at 2.5 × 10^{-6} M (A), 5.0 × 10^{-6} M (B), 1.0 × 10^{-5} M (C), and 2.0 × 10^{-5} M (D) in cyclohexane/acetone 98:2. [(S)-**6**]/[**7**] = 1:2; λ_{ex} = 363 nm, em/ex slit widths = 1.5/3 nm.

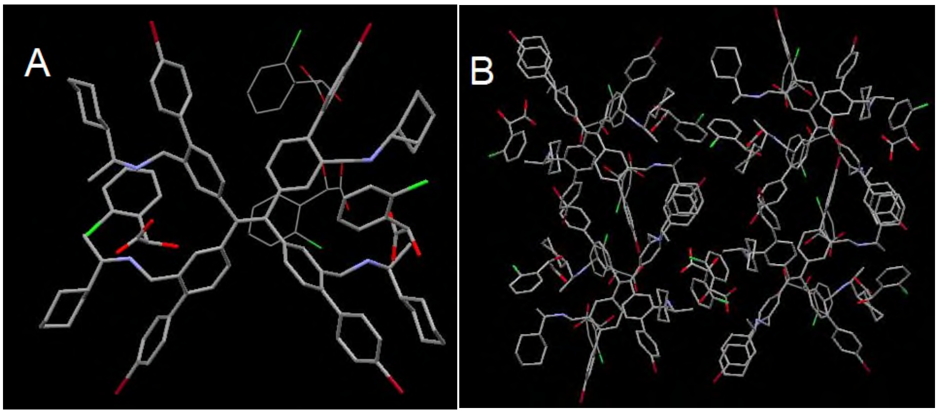

Supplementary Figure 43. Crystal structure (A) of (*S*)-**6**-(*R*)-**16** complex grown from $CHCl_3$/acetone/acetonitrile and the molecules packing (B) (hydrogen atoms were omitted for clarity).

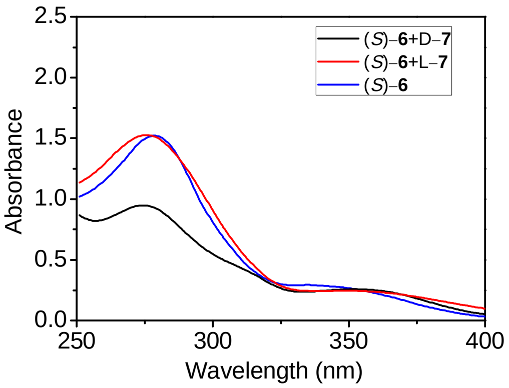

Supplementary Figure 44. UV–vis spectra of (*S*)-**6** and di-P-toluoyl-(D or L)-tartaric acid mixed solution in cyclohexane ([(*S*)-**6**] = 1/2 [Acid] = 1 x 10^{-5} M).

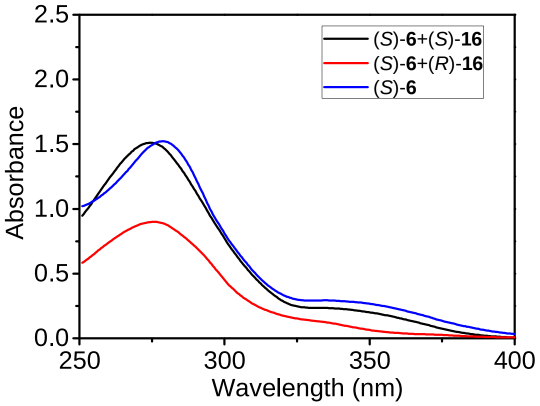

Supplementary Figure 45. UV–vis spectra of (*S*)-**6** and (R or S)-2-Chloromandelic acid mixed solution in cyclohexane ([(*S*)-**6**] = 1/4 [**16**] = 1 x 10^{-5} M).

Supplementary Tables

Supplementary Table 1. The fluorescence quantum yield (Φ%)[a]

Compound	Solvent	Φ%	Fluorescence Lifetime
(*R*)-**6**	THF	1.55%	
(*S*)-**6**	THF	1.61%	
(*R*)-**6**	H_2O/THF 95:5	50.8%	
(*S*)-**6**	H_2O/THF 95:5	52.6%	
(*R*)-**6**	Acetone	0.72%	
(*S*)-**6**	Acetone	0.79%	
(*R*)-**6**	Cyclohexane/Acetone 98:2	12.8%	4.27 ns
(*S*)-**6**	Cyclohexane/Acetone 98:2	12.2%	3.99 ns
(*S*)-**6 +** 1eq D-**7**	Cyclohexane/Acetone 98:2	24.4%	
(*S*)-**6 +** 1eq L-**7**	Cyclohexane/Acetone 98:2	25.6%	
(*S*)-**6 +** 2eq D-**7**	Cyclohexane/Acetone 98:2	29.7%	3.58 ns
(*S*)-**6 +** 2eq L-**7**	Cyclohexane/Acetone 98:2	31.9%	4.44 ns
(*S*)-**6 +** 4eq D-**7**	Cyclohexane/Acetone 98:2	44.1%	
(*S*)-**6 +** 4eq L-**7**	Cyclohexane/Acetone 98:2	49.8%	
(*S*)-**6 +** 2eq D-**7**	Acetone	23.2%	
(*S*)-**6 +** 2eq L-**7**	Acetone	26.7%	
(*S*)-**6 +** 1eq (*S*)-**16**	Cyclohexane/Acetone 98:2	12.2%	
(*S*)-**6 +** 1eq (*R*)-**16**	Cyclohexane/Acetone 98:2	15.3%	
(*S*)-**6 +** 2eq (*S*)-**16**	Cyclohexane/Acetone 98:2	11.2%	
(*S*)-**6 +** 2eq (*R*)-**16**	Cyclohexane/Acetone 98:2	14.1%	
(*S*)-**6 +** 4eq (*S*)-**16**	Cyclohexane/Acetone 98:2	8.9%	2.15 ns
(*S*)-**6 +** 4eq (*R*)-**16**	Cyclohexane/Acetone 98:2	9.9%	1.22 ns

[a] The fluorescence quantum yield was measured using quinine sulfate (Φ_f = 0.546) in 0.5 M H_2SO_4 as standard.
